# The Development and Evaluation of a Literature-Based Dietary Index for Gut Microbiota

**DOI:** 10.3390/nu16071045

**Published:** 2024-04-03

**Authors:** Bezawit E. Kase, Angela D. Liese, Jiajia Zhang, Elizabeth Angela Murphy, Longgang Zhao, Susan E. Steck

**Affiliations:** 1 Department of Epidemiology and Biostatistics, Arnold School of Public Health, University of South Carolina, Discovery 1, 915 Greene Street, Columbia, SC 29208, USA; bkase@email.sc.edu (B.E.K.);; 2 Department of Pathology, Microbiology and Immunology, School of Medicine Columbia, University of South Carolina, Columbia, SC 29208, USA

**Keywords:** gut microbiota, dysbiosis, diet, dietary index, dietary index for gut microbiota, DI-GM

## Abstract

The aim of the study was to develop and evaluate a novel dietary index for gut microbiota (DI-GM) that captures dietary composition related to gut microbiota profiles. We conducted a literature review of longitudinal studies on the association of diet with gut microbiota in adult populations and extracted those dietary components with evidence of beneficial or unfavorable effects. Dietary recall data from the National Health and Nutrition Examination Survey (NHANES, 2005–2010, *n* = 3812) were used to compute the DI-GM, and associations with biomarkers of gut microbiota diversity (urinary enterodiol and enterolactone) were examined using linear regression. From a review of 106 articles, 14 foods or nutrients were identified as components of the DI-GM, including fermented dairy, chickpeas, soybean, whole grains, fiber, cranberries, avocados, broccoli, coffee, and green tea as beneficial components, and red meat, processed meat, refined grains, and high-fat diet (≥40% of energy from fat) as unfavorable components. Each component was scored 0 or 1 based on sex-specific median intakes, and scores were summed to develop the overall DI-GM score. In the NHANES, DI-GM scores ranged from 0–13 with a mean of 4.8 (SE = 0.04). Positive associations between DI-GM and urinary enterodiol and enterolactone were observed. The association of the novel DI-GM with markers of gut microbiota diversity demonstrates the potential utility of this index for gut health-related studies.

## 1. Introduction

Gut microbiota play a crucial role in human health, including the immune system, metabolic regulation, and neurobehavioral traits [1,2,3]. Imbalance in gut microbiota or dysbiosis is linked with insulin resistance, increased trimethylamine N-oxide production, colonic cell proliferation, and other disease pathways [1]. Dysbiosis is characterized by lower bacterial diversity, lower species richness, and lower relative abundance of beneficial bacteria, leading to lower production of short-chain fatty acids (SCFAs), mainly acetate, propionate, and butyrate [1]. Diet is among the main factors that strongly influence gut microbiota composition [4,5,6]. 

There is a growing interest in manipulating gut microbiota through diet. Studies have shown that changes in diet can induce shifts in the species composition of the gut microbiota [1,7]. Healthy microbiota, such as butyrate-producing bacteria, have been shown to increase with higher dietary fiber intake [8]. A review that included 19 human intervention studies reported that fermented foods may be potential dietary targets to prevent or overcome gut dysbiosis in humans [9]. Other studies examined the effects on gut microbiota for diets that are characterized by a reduction in or exclusion of a specific nutrient from a dietary pattern, such as ketogenic diets, gluten-free diets, and vegan diets [6,10]. However, there is a lack of a comprehensive measure of diet or a dietary index that can quantify individuals’ diets in terms of attaining a healthy gut microbiota.

Dietary indices are tools used to characterize ways of eating based on dietary assessment data [11,12]. Among the most commonly used currently available indices are the Healthy Eating Index (HEI), the alternate HEI (aHEI), the Mediterranean Diet Score (MDS), and the Dietary Approaches to Stop Hypertension (DASH) [13]. Although these indices are useful in examining the relationship between diet quality and health outcomes [13], their associations with gut microbiota diversity and richness indicators have been inconsistent [5,14,15]. 

Developing a tool that can measure diet quality associated with maintaining healthy gut microbiota is essential not only for understanding how dietary change can modify gut microbiota but ultimately for designing dietary interventions to alleviate dysbiosis-related diseases. To construct a literature-derived dietary index for gut microbiota (DI-GM), we conducted a systematic review of interventional and longitudinal observational studies that assessed the association of different foods or food groups on gut microbiota composition in adults. The novel DI-GM was then compared to existing dietary indices based on its strength in association with indirect biomarkers of gut microbiota diversity using data from the National Health and Nutrition Examination Survey (NHANES). Urinary enterodiol and enterolactone are indirect biomarkers of gut microbiota diversity. We expected a positive association between the DI-GM and levels of urinary enterodiol and enterolactone indicating enhanced gut microbiota diversity.

## 2. Materials and Methods

### 2.1. Development of the DI-GM 

#### 2.1.1. Literature Search Strategy

The literature search and reporting were conducted following the Preferred Reporting Items for Systematic Reviews and Meta-analysis guidelines (PRISMA) [16]. Articles published in peer-reviewed journals were identified by systematic search in PubMed and Embase databases by two authors (primary investigator and librarian). The search terms included a combination of ‘gut’, ‘microbiota’, ‘dysbiosis’, ‘alpha diversity’, ‘short-chain fatty acid’, ‘*Bifidobacterium*’, ‘*Firmicutes*’, ‘food’, ‘diet’, ‘nutrient’, and equivalent terms. The results were filtered for articles published in the English language, human species, and publication date since January 2008, representing a timeframe when current sequencing technologies to study gut microbiota became available. The last search was run on 17 October 2021, and resulted in 19,306 articles. The complete list of search terms and search strategies for each database can be found in Supplementary Material Section S1.

#### 2.1.2. Selection Criteria

Articles retrieved from the initial search were exported to Covidence to remove duplicates, conduct title and abstract screening, and subsequently full-text review. Studies that examined the effect of certain foods or food groups on gut microbiota composition were the focus of the review. Eligibility criteria to select articles are presented in Table 1. Articles were included if they met inclusion criteria for the study population [adult participants, i.e., age 20 years and above and without inflammatory bowel disease (IBD)], exposure/intervention (at least one food, food group, or nutrient), outcome (such as gut microbiota richness and evenness indices, phyla count, levels of SCFAs, a ratio of *Firmicutes* to *Bacteroidetes*, or any specific gut bacteria), and study design (either intervention studies including randomized controlled studies, non-randomized interventions, and cross-over studies, or longitudinal observational studies). Studies that examined extracts, prebiotics, probiotics, or overall diet quality indices were excluded.

#### 2.1.3. Data Extraction

One author extracted relevant data from all eligible articles, and a second author checked that the extracted data were consistent with the reported findings in the articles. Data extraction was conducted using a template in Excel, including first author and year of publication, description of the study population (i.e., sample size, age, sex), study design and length of follow-up, food or food group (type, amount, and duration of consumption and comparison arm), and effect on gut microbiota (effect estimate, *p*-value, summary of the finding). Risk-of-bias assessments were performed using design-specific tools: Risk of Bias for Nutrition Observational Studies tool (RoB-NObs) [17], and Cochrane risk-of-bias tool for randomized parallel-group trials, cluster-randomized trials, and crossover trials [18]. 

#### 2.1.4. Data Synthesis

The associations of foods or food groups with gut microbiota were summarized descriptively in terms of evidence of beneficial, unfavorable, or no observed effect. Attributes of gut microbiota (outcome) that were of interest in this review were diversity indices (α-diversity and β-diversity indices), fecal SCFA levels, change in ratios of phyla, and change in specific bacteria (relevant bacteria in disease mechanisms). A comprehensive list of gut microbiota outcomes can be found in Supplementary Material Section S2. Beneficial effects on gut microbiota were defined as an increase in α-diversity and β-diversity indices; an increase in total SCFA, butyrate, acetate, propionate, or isobutyrate; or balanced *Firmicutes*/*Bacteroidetes* ratio [19,20,21,22]. For specific bacteria, a beneficial effect was defined as an increase in *Faecalibacterium*, *Bifidobacterium*, *Lactobacillus*, *Lactococcus*, *Parabacteroides*, *Roseburia*, *Eubacterium rectale*, *Eubacterium hallii*, *Akkermansia*, *Akkermansia muciniphila*, *Prevotella*, *Prevotella copri*, *Anaerostipes*, *Anaerostipes hadrus*, *Veillonellaceae*, *Parabacteroides distasonis*, *Gemmiger*, or *Moraxellaceae* [19,20,21,22]. A decrease in *Bacteroides*, *Bacteroides fragilis*, *Fusobacteria*, *Streptococcus*, *Clostridium*, *Clostridium symbiosum*, *Clostridium perfringens*, *Dialister*, *Alistipes*, *Bilophila*, *Ruminococcus gnavus*, *Dorea*, *Actinomyces*, *Odoribacter*, *Blautia*, *Lachnospira*, *Lachnospiraceae*, *Sutterella*, *Enterobacteriaceae*, or *Klebsiella* sp., were also indicators of beneficial effect on gut microbiota [19,20,21,22]. Findings of opposite effects to those defined as beneficial effects were considered unfavorable effects. 

Given the high variability of dose and duration of consumption of foods or food groups and variation in reporting type of effect measures across studies, we did not meta-analyze the data and no quantitative summary measures were computed. Consensus on the beneficial or unfavorable effects of a specific food or food group was reached if at least one intervention study or more than two observational studies showed supporting evidence with minimal risk of bias and no conflicting evidence from other studies included in the review. Based on this, foods or food groups with evidence of a beneficial or unfavorable effect on gut microbiota were selected to be components of the DI-GM. 

### 2.2. Evaluation of the DI-GM

#### 2.2.1. Study Population

The novel DI-GM was first computed using dietary data from NHANES. NHANES is a comprehensive population-based survey designed to collect data on the diet, nutritional status, health, and health behaviors of the US civilian population per 2-year cycle [23]. NHANES incorporated urinary enterolignan assessment, indirect biomarkers of gut microbiota diversity [24], in 6 cycles starting from 1999–2010.

For the current study, data from 3 cycles, 2005–2006, 2007–2008, and 2009–2010 of NHANES were used to examine the association of diet (indicated by the novel DI-GM, HEI, and MDS) and urinary enterolignans (*n* = 31,034). These three cycles were selected because a similar dietary assessment method, 24-h dietary recall, was used across the cycles in addition to the assessment of urinary enterolignan. We further excluded participants aged less than 20 years, who did not complete two days of 24-h dietary recall, who reported extreme daily caloric intake (less than 500 kcals or greater than 6000 kcals), who had extreme body mass index (BMI, less than 15 kg/m^2^ or greater than 65 kg/m^2^), or those with incomplete covariate data. We included 11,982 participants for descriptive analyses and correlation analyses of the three dietary indices. In the final analysis to examine the association of the dietary indices and urinary enterolignan, participants with missing data on urinary enterolignan were also excluded, resulting in the final analytic sample of *n* = 3812.

#### 2.2.2. Dietary Assessment 

In NHANES, two interviewer-based 24-h dietary recall assessments using USDA’s Automated Multiple-Pass Method were conducted 3–10 days apart [25]. The first 24-h dietary recall was conducted in Mobile Examination Centers (MEC), and the second 24-h dietary recall was obtained by telephone interview [25]. Portion size estimations were performed using a standard set of measuring guides. Codes to all foods and beverages and amounts reported by participants during their 24-h dietary interviews were assigned using the food composition database of the USDA’s Food and Nutrient Database for Dietary Studies (FNDDS) [25]. For the current study, the mean intake of foods, food groups, and nutrients from the two 24-h recalls were used to construct the DI-GM and existing indices (HEI-2015 and MDS).

Details about the construction of the DI-GM are presented in the Results section. The HEI-2015 assesses conformity with the 2015 Dietary Guidelines for Americans [26]. The HEI-2015 has 13 components and the total score ranges from 0 to 100, with higher scores indicating a healthier diet. The MDS is an index that quantifies conformity to the traditional Mediterranean diet [27]. The MDS has nine components, and scores range from 0 to 9 with higher scores indicating better conformity to the Mediterranean diet.

#### 2.2.3. Urinary Enterolignans Assessment

Urinary enterolignans (enterodiol (ng/mL) and enterolactone (ng/mL)) were measured in spot urine samples. Detailed specimen collection and processing instructions are discussed in the NHANES Laboratory/Medical Technologists Procedures Manual [28]. Urinary enterolignan concentrations were normalized by urinary creatinine (mg/dL) to correct for urine dilution (expressed as ug/g creatinine) [29]. 

#### 2.2.4. Statistical Analysis 

Sample characteristics of the unweighted total sample (*n* = 11,982) were presented using mean (standard error) for continuous variables and frequency (percentage) for categorical variables. Correlations between the DI-GM and pre-existing indices (HEI-2015 and MDS) were determined using the Pearson correlation coefficient. Bivariate and multivariable linear regression were used to examine associations between DI-GM and urinary enterolignans in the smaller subsample with urinary biomarker data (*n* = 3812). In the multivariable linear regression models, sex (female, male), age (in years), race and ethnicity (Hispanic, non-Hispanic Black, non-Hispanic White, other), education level (<12th grade, high school diploma, some college education, college graduate and above), marital status (married, widowed, divorced or separated, living with a partner, never married), smoking status (never smoked, occasional smoker, regular smoker, previous smoker), alcohol use in the past 12 months (none, once or more per month), and BMI (kg/m^2^) were included to adjust for potential confounding effects. Similar bivariate and multivariable linear regression analyses were used to examine the association between existing indices (HEI-2015 and MDS) and urinary enterolignans.

All analyses were run using SAS survey procedures considering the NHANES strata, cluster, and sampling probability weights. We confirmed the assumption of normality was not violated for any of the variables using histograms and Kolmogorov–Smirnov tests. Both crude and adjusted regression coefficients (β) with a 95% confidence interval (CI) were reported. All analyses were performed using SAS^®^ 9.4 software.

## 3. Results

### 3.1. Construction of the DI-GM

**Figure 1:**
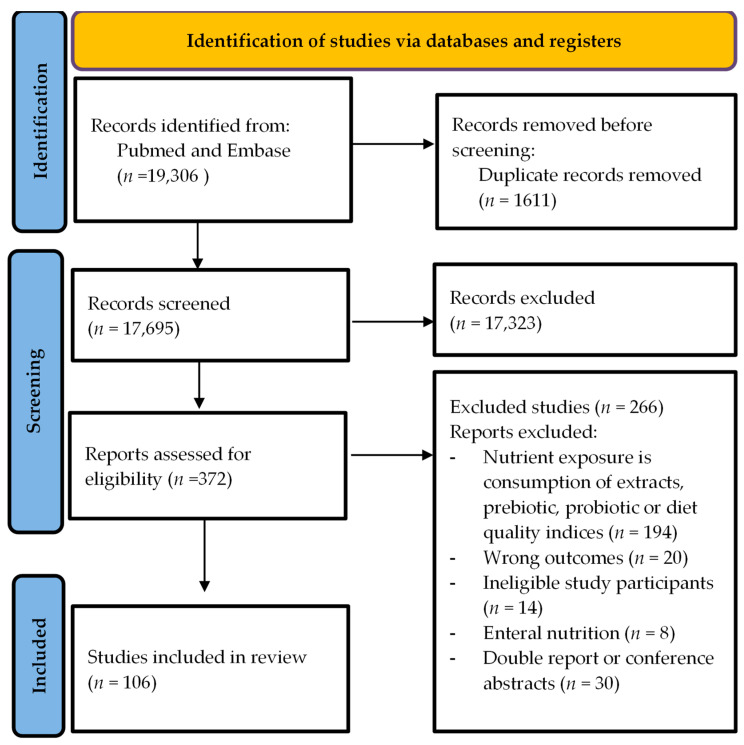
Prisma flow chart.

A total of 106 articles were included in the systematic review, composed of intervention studies (*n* = 102) and longitudinal observational studies (*n* = 4). Figure 1 shows the PRISMA flow chart. The articles were grouped by the type of food examined, i.e., articles on dairy, meat and fish products, legumes and nuts, grains, fiber, fruits, vegetables, macronutrients, oils and seasonings, coffee and tea, and alcohol and wine. Supplementary Table S1 shows a summary table of the findings from the reviewed articles.

Among the articles in the dairy group, five articles were intervention studies [30,31,32,33,34,35,36], and one was a prospective cohort study [37]. The articles examined the effect of intake of total dairy, kefir, yogurt, and whole milk on gut microbiota diversity and composition, SCFA levels, and specific bacteria count. Kefir and fermented dairy intake were associated with beneficial changes in gut microbiota, mainly an increase in *actinobacteria* [30]. Dairy intake was associated with some beneficial effects such as an increase in *Faecalibacterium* and *Bifidobacterium* and some unfavorable effects such as an increase in *Streptococcus* and *Clostridium* [33,37]. Based on the consistency of evidence and the number of studies, fermented dairy was included as a component of the DI-GM, but the evidence was too limited for other dairy products at this time, so they were not included as components of the index.

There were seven articles in the meat and fish products group, and all were intervention studies [38,39,40,41,42,43]. Foods examined in these articles were sardines, salmon, cod, red meat, and animal-based diets. Animal-based diet was associated with an increase in the abundance of bile-tolerant microorganisms (*Alistipes*, *Bilophila*, and *Bacteroides*) [42]. Findings on sardines, salmon, and cod intake were inconclusive, and thus were not included in the DI-GM [38,40]. The review indicates sufficient evidence to support the inclusion of red and processed meat as unfavorable components of the DI-GM.

Among the articles in the legumes and nuts group, 14 were intervention studies [44,45,46,47,48,49,50,51,52,53,54,55,56,57], and one was a cohort study [58]. Foods examined in these articles were flaxseed, almonds, pistachios, walnuts, chickpeas, soy, and total legume intake. Findings on almond intake indicate some beneficial effects such as an increase in bacterial richness and evenness [46], and decrease in *Bacteroides fragilis* [49], and some unfavorable effects such as a decrease in *Actinobacteria* and *Bifidobacterium* [48]. Intake of chickpeas was associated with an increase in *Faecalibacterium prausnitzii* and a decrease in *Clostridium* clusters but no effect on α-diversity and SCFA levels [52]. Intake of soy was associated with an increase in *Bifidobacteria* and *Lactobacilli* and a decrease in *Clostridia* [55,56]. The evidence supports the inclusion of chickpeas and soy as components of the DI-GM; however, the evidence was inconclusive for flaxseed, almonds, pistachios, and walnuts.

A total of 20 articles, all of which were intervention studies, were focused on the effects of intake of grains on gut microbiota [59,60,61,62,63,64,65,66,67,68,69,70,71,72,73,74,75,76,77,78]. The foods examined in these articles were whole grains, refined grains, rye, oatmeal, barley, brown rice, and ancient grains (“Tim-ilia”, “Margherito”, and “Russello”). Whole-grain intake was associated with an increase in *Bifidobacterial*, *Prevetolla*, and *Roseburia hominis* [59,60,62]. Compared to intake of refined grains, whole grain intake was associated with an increase in acetate and total SCFA [68]. The accumulation of evidence from intervention studies supported the inclusion of whole grains and refined grains as components of the DI-GM.

A total of three intervention studies examined intake of fiber [7,79,80]. Intake of fiber was associated with an increase in *Firmicutes*, *Bifidobacterium*, *E. rectale*, and an increase in fecal butyrate [7,79]. Evidence from the reviewed intervention studies supported the inclusion of fiber as a component of the DI-GM.

Among articles that examined intake of fruits, 18 were intervention studies [81,82,83,84,85,86,87,88,89,90,91,92,93,94,95,96,97,98], and one was a prospective cohort study [99]. Fruits examined in these articles were olives, mangos, apples, cranberries, orange juice, dates, strawberries, avocados, boysenberry juice, raisins, and total fruit and vegetable intake. Intake of cranberries was associated with an increase in *Bacteroidetes* and a decrease in *Firmicutes* [84,85]. No effect on α-diversity or β-diversity, SCFA, or fecal microbiota was found after intake of dates [88], strawberries [89], and boysenberry juice beverage [96]. Intake of avocados was associated with an increase in *Faecalibacterium*, *Veillonellaceae*, and *Prevotellaceae* and an increase in fecal acetate in two intervention studies [91,92]. Among the fruits that were examined in the reviewed studies, evidence was present for only cranberries and avocado to suffice inclusion as components of the DI-GM. 

A total of ten intervention studies were included in the vegetable group, which examined the effects of green leafy vegetables [100], kimchi [101,102], inulin-type fructans-rich vegetables [103], broccoli [104,105,106], cruciferous vegetables [107], tomato and carrot juice [108], and ginger juice [109]. Inulin-type fructans-rich vegetables include artichokes, asparagus, chicory root, garlic, and onions [110]. No changes in diversity indices or phyla were found after intake of green leafy vegetables [100], tomato or carrot juice [108]. Intake of kimchi was associated with some beneficial effects such as an increase in Chao1 Richness index and Shannon index, and an increase in *Actinobacteria*, *Bacteroides*, and *Prevotella*, and some unfavorable effects such as a decrease in *Roseburia* and *Bifidobacterium* [101,102]. Intake of steamed broccoli was associated with an increase in *Bacteroidetes* phylum and *Bacteroides* genus, a decrease in sulphate-reducing bacteria, and an increase in the ratio of *Bacteroidetes* to *Firmicutes* [104,105]. The evidence for broccoli warranted the inclusion as a component of the DI-GM; however, the evidence for other vegetables was inconclusive. 

Among articles that examined the effect of macronutrients on gut microbiota, 13 were intervention studies [111,112,113,114,115,116,117,118,119,120,121,122,123], and one was a prospective cohort study [124]. A high protein diet was associated with a decrease in *Faecalibaculum*, *Prevotella_2*, and *Lachnospiraceae_UCG-004* [111,115]. A high-fat diet was associated with a decrease in *Firmicutes* and lower total bacterial count [117,119]. Three studies found no association between fat intake and α-diversity and β-diversity [118,120,121]. Among the macronutrients examined, high-fat was included as a component of the DI-GM. 

Articles that examined oils and seasonings were five intervention studies [125,126,127,128,129]. Oils and seasonings examined in these articles were artificial sweeteners, polyphenol-rich mixed spices, soybean oil, extra virgin olive oil, and coconut oil. No change in richness and evenness, SCFA, and bacteria phyla were found after intake of the examined oils and seasonings [126,128,129]. Thus, oils and seasonings were not included as components of the DI-GM. 

The effects of coffee and tea intake were examined using three intervention studies [130,131,132]. Intake of coffee was associated with an increase in *Prevotella* and *Bifidobacterium* and a decrease in *Bacteroidetes* [130,131]. Intake of green tea was associated with an increase in α-diversity and β-diversity, and an increase in *Firmicutes*, *Actinobacteria*, and *Bifidobacterium* [132]. Both coffee and green tea were included as components of the DI-GM.

Two intervention studies examined the effect of red wine and gin on gut microbiota composition [133,134]. An increase in *Firmicutes*, *Bacteroidetes*, and *Fusobacteria* was found after red wine intake in one study [134]. Another study reported no change in α-diversity and *Bacteroidetes*/*Firmicutes* ratio after wine consumption [133]. We did not find sufficient evidence for red wine and gin, thus neither were included as components of the DI-GM. 

Based on the reviewed articles, 14 foods and nutrients were identified as having beneficial or unfavorable effects on gut microbiota (Table 2). Beneficial effects were an increase in α-diversity and β-diversity indices; an increase in total SCFA, butyrate, acetate, propionate, or isobutyrate; or balanced *Firmicutes*/*Bacteroidetes* ratio. Beneficial components identified were fermented dairy, chickpeas, soybean (including tofu), whole grains, fiber, cranberries, avocados, broccoli, coffee, and green tea. Unfavorable effects on gut microbiota were findings of opposite effects to those defined as beneficial effects. Unfavorable components identified were red meat, processed meat, refined grains, and a high-fat diet (≥40% energy from fat). These foods and nutrients were included as components of the novel DI-GM. To score the DI-GM, sex-specific median intakes of each component were computed except for a high-fat diet for which a fixed cutoff, i.e., 40% energy from fat, was used. A score of 1 is assigned for participants who consumed above the sex-specific median for each beneficial component and for participants who consumed below the sex-specific median for each unfavorable component. A score of 0 is assigned for participants who consumed below the sex-specific median for each beneficial component and for participants who consumed above the sex-specific median for each unfavorable component. The scores for each component are summed to obtain the DI-GM score ranging from 0–14. A higher DI-GM score indicates a healthier gut microbiota.

### 3.2. Correlations between the DI-GM and Markers of Gut Microbiota Diversity

The DI-GM was computed using 24-h dietary recall data in NHANES. The mean age of participants was 47.3 years (±0.4), and about half of the participants (51.8%) were female. Most participants were non-Hispanic White (72.1%) and were married (59.1%) (Table 3).

The DI-GM scores in NHANES range from 0–13 with a mean ± standard error (SE) of 4.80 (±0.04). The green tea component was not included in the scoring of the DI-GM in NHANES because the specific type of tea consumption was not recorded in the 24-h dietary recall data. The correlation between DI-GM and HEI-2015 was 0.54 (*p* < 0.0001), and the correlation between DI-GM and MDS was 0.42 (*p* < 0.0001), as shown in Table 4.

The DI-GM was modestly positively correlated with both creatinine-adjusted enterodiol (r = 0.19, *p* < 0.0001) and enterolactone (r = 0.22, *p* < 0.0001). Of the 13 DI-GM components, 10 components were correlated to the urinary enterolignans. Fermented dairy, red meat, processed meat, refined grains, and high-fat diet were negatively correlated with creatinine-adjusted enterodiol and enterolactone levels (Table 5), while intake of chickpeas, soybean, whole grains, fiber, avocados, broccoli, and coffee were each positively correlated with creatinine-adjusted enterodiol and enterolactone concentrations. 

A one-unit increase in DI-GM was associated with an increase by 0.12 μg/g (95% CI: 0.08, 0.17) in creatinine-adjusted enterodiol concentrations and 0.14 μg/g (95% CI: 0.09, 0.18) in creatinine-adjusted enterolactone concentrations in the multivariable model (Table 6).

A one-unit increase in the rescaled HEI-2015 (rescaled scores range 0–10) was associated with an increase of 0.21 μg/g (95% CI: 0.16, 0.26) in creatinine-adjusted enterodiol concentrations and 0.20 μg/g (95% CI: 0.15, 0.26) in creatinine-adjusted enterolactone concentrations in the multivariable model. A one-unit increase in MDS was associated with an increase of 0.11 μg/g (95% CI: 0.07, 0.15) in creatinine-adjusted enterodiol and by 0.12 μg/g (95% CI: 0.08, 0.17) in creatinine adjusted enterolactone.

## 4. Discussion

We developed a novel dietary index, DI-GM, from an extensive literature review and showed that the DI-GM was associated with indirect biomarkers of gut microbiota diversity. The DI-GM is composed of 14 foods or nutrients: fermented dairy, chickpeas, soybean, whole grains, fiber, cranberries, avocados, broccoli, coffee, and green tea were beneficial components, and refined grains, red meat, processed meat, and high-fat diet (≥40% energy from fat) were unfavorable components. Selection of the components of the DI-GM was based on having either beneficial or unfavorable effects on gut microbiota indicated by changes in gut microbiota diversity indices, level of SCFA production, or increase in the count of specific bacteria. The demonstration of an association between the DI-GM and biomarkers of gut microbiota diversity in NHANES indicates the construct validity of the DI-GM to measure the quality of diet in relation to gut microbiota diversity.

The focus and development of the DI-GM make it unique compared to pre-existing indices that were developed to demonstrate how diet modulates gut microbial composition [5,14,135,136,137]. The DI-GM is literature-derived and focuses on broad attributes of gut microbiota including diversity indices, production of SCFA, change in phyla, and specific bacteria. Among related pre-existing indices is the sulfur-metabolizing diet score where dietary constituents associated with the enrichment of sulfur-metabolizing bacteria were identified using clustering and regression techniques [135,136]. The components of the sulfur-metabolizing diet score include processed and red meat, liquor, low-calorie drinks, beer, fruit juice, legumes, mixed (other) vegetables, and sweets/desserts [135,136]. There are some similarities between the DI-GM and the sulfur-metabolizing diet score because sulfur-metabolizing bacteria were among the gut bacteria of interest in the construction of the DI-GM. Some differences between the DI-GM and sulfur-metabolizing diet likely arose due to the methods used in creating these indices/patterns. The DI-GM was developed based on current literature, whereas the sulfur-metabolizing diet score is data-driven within a specific study population (the Health Professionals Follow-up Study) [135], and replicated in one other study population (Nurses’ Health Study) [136]. In addition, the target for the DI-GM is broader in which diversity indices, production of SCFA, change in phyla, and specific bacteria were outcomes compared to the target for the sulfur-metabolizing diet which focused on only sulfur-metabolizing bacterium. Thus, the DI-GM can be utilized in studies where the focus is not limited to sulfur-metabolizing bacterium.

While the novel DI-GM is intended to measure diet quality in relation to gut microbiome health, it appears to also inherently measure the overall healthfulness of diet as evidenced by the DI-GM showing a correlation, albeit moderate, with the HEI-2015 and MDS. The strength of the diet index–urinary enterolignan association using DI-GM was slightly weaker than using HEI-2015 but was similar to using the MDS. This may be due to the inclusion of broad sources of dietary lignans in the HEI-2015 which are not included in the DI-GM. Both indices include whole grains which are good sources of lignans, but the HEI-2015 includes additional lignan sources such as legumes, fruits, and vegetables. An increase in enterolignan excretion has been shown to be explained by higher gut bacterial diversity and composition as well as dietary intake of lignans [24]. Although the association between the DI-GM and urinary enterolignans is in the expected direction, there are no threshold levels of urinary enterolignans that mark levels of gut bacteria diversity and community structure to speculate on the strength of association. 

Some dietary components overlapped between the DI-GM, HEI-2015, and MDS, for instance, whole and refined grains. The DI-GM like the HEI-2015 and MDS is an a priori index that can be used to compare dietary patterns of different populations. Given the overarching role of gut microbiota in different diseases, the DI-GM can be used to examine diet-disease associations similar to the HEI-2015 and MDS. However, the DI-GM differs from existing indices in that for some components, it includes specific foods, rather than a food group [26,138]. Most of the reviewed articles in the construction of the DI-GM were dietary intervention studies that focused on the provision of a specific food. The inclusion of specific foods in the DI-GM may be potentially beneficial in determining dietary recommendations. 

One of the unique dietary components of the DI-GM is fermented dairy. There is increasing evidence to support the role of fermented foods in enhancing the gut microbiota. Fermented dairy such as yogurt, cheese, and kefir contain lactic acid bacteria and lactic acid and have been shown to increase the abundance of *Lactobacillus* spp. counts in the gut, which has the potential to overcome gut dysbiosis [9,139]. However, we found a negative correlation between fermented dairy intake and indirect biomarkers of gut microbiota diversity in NHANES. This finding could be due to the added sugar or fat content in some fermented dairy products. It has been previously shown that the microbial profile associated with the intake of yogurt differs between natural and sweetened yogurt [140]. In the United States, the consumption of sweetened yogurt far exceeds the consumption of natural yogurt [141]. We speculate that combining natural and sweetened yogurt as one component in the DI-GM could mask the positive effect of natural yogurt and emphasize the negative effect of sweetened yogurt as it is predominantly consumed. However, in constructing the DI-GM we did not have sufficient evidence from the literature to distinguish the types of yogurt in the fermented dairy component of the DI-GM.

Chickpeas and soybeans were among the beneficial components of the DI-GM. These components include soybean oligosaccharides and raffinose that selectively enhance the growth of *Bifidobacterium*, a bacteria that has shown anti-cancer properties [52,55,142]. Broccoli is also among the beneficial components. Similar finding from the sulfur-metabolizing diet indicates that plant-based sulfur sources, such as those found in legumes and vegetables, are associated with the relative depletion of sulfur-metabolizing bacteria [135]. Glucosinolates in broccoli are degraded by gut bacteria into phytochemicals that decrease the initiation and progression of cancer [143]. It has been shown that whole grains and vegetable consumption enhance gut microbes known to utilize components of the whole grain, including fiber, and subsequently produce a range of short-chain fatty acids [137]. Both coffee and green tea were beneficial components in the DI-GM, and their polyphenol content may explain this [144,145]. Polyphenols in coffee, mainly chlorogenic acids, stimulate the growth of *Bifidobacteria* spp. and a decrease in *Clostridium* spp. [144]. Similarly, green tea catechins (phenolic compounds) have been shown to act as prebiotics by stimulating specific bacteria that metabolize these compounds [145]. 

Red meat, processed meat, and a high-fat diet were among the unfavorable components of DI-GM, and studies suggest different mechanisms through which these foods bring about dysbiosis. A high-fat diet has been linked to reduced microbial count and an increase in gut permeability [146]. High-fat diets enriched with meat-based proteins may promote distinct and less diverse populations of sulfur-metabolizing bacteria due to their high sulfur content from both sulfur-containing amino acids and the organic sulfurs found in preservatives [135]. In addition, dietary choline or L-carnitine found in red and processed meat has been linked to gut microbiota responsible for the biosynthesis of Trimethylamine N-oxide (TMAO), a pro-atherosclerotic metabolite [147,148]. More research is needed to identify the effect of other factors related to meat consumption such as methods of cooking on gut microbiota diversity [149].

The study has some limitations. Although a substantial number of articles (*n* = 106) were included in the review, there was a limited number of articles per food or food group. Thus, the selection of the components of the DI-GM relied on limited articles per food, and foods that have not been studied in relation to gut microbiota were not included in the index. Therefore, the DI-GM may benefit from revisions as more evidence becomes available. Variations between articles in reporting consumption levels of foods and methods of cooking made it difficult to add such attributes to the components of the DI-GM. Another limitation was the lack of a clear definition of what constitutes healthy/unhealthy gut microbiota and the variation in ways that articles report markers of healthy/unhealthy gut microbiota. To overcome this, we constructed a comprehensive list of direct markers of gut microbiota outcomes, i.e., diversity indices, levels of SCFA, and specific bacteria. The use of urinary enterolignans as indirect biomarkers of gut microbiota diversity has a limitation as it is a non-specific indicator of gut microbiota diversity. Thus, the study needs to be replicated in a dataset where direct gut microbiota diversity measures are available. 

The study has strengths that are worth noting. The DI-GM is derived based on a review of longitudinal studies, most of which were intervention studies and thus provided the highest level of causal evidence for the associations under study. Given the DI-GM is an a priori-defined index, it can be computed using dietary intake data of existing studies, allowing more direct comparisons across studies when examining DI-GM–health outcome association [150]. Most of the DI-GM components are foods as opposed to nutrients which can more easily be interpreted for dietary recommendations. Lastly, we showed that the DI-GM has construct validity in measuring the role of diet in gut microbiota diversity using a nationally representative sample. 

## 5. Conclusions

In conclusion, we constructed a novel dietary index, i.e., DI-GM, based on a literature review that characterizes the relationship between diet and different aspects of gut microbiota. Beneficial components included fermented dairy, chickpeas, soybean, whole grains, fiber, cranberries, avocados, broccoli, coffee, and green tea, while unfavorable components included refined grains, red meat, processed meat, and greater than 40% of daily energy from fat. We found that the DI-GM was positively associated with urinary enterolignans, indicating a relationship with gut microbiota diversity. Future studies that incorporate gut microbiome data are needed to evaluate the utility of the index.

## Figures and Tables

**Table 1:** Inclusion and exclusion criteria for articles in the systematic review.

Inclusion Criteria	Exclusion Criteria
Study population: age 20 years and older	Participants with gastrointestinal disorders (irritable bowel syndrome, inflammatory bowel diseases, colorectal cancer) Participants on enteral nutrition
Exposure/Intervention: foods and food groups	Exposure/Interventions such as extracts, prebiotics, probiotics, supplements, and diet quality indices
Outcomes such as gut microbiota diversity indices, SCFAs, ratio of *Firmicutes* to *Bacteroidetes* or any specific gut bacteria	
Study designs: Intervention studies and longitudinal observation studies	

SCFAs: short-chain fatty acids.

**Table 2:** Components of the DI-GM identified based on the systematic review.

Component	Included Foods within the Component	Scoring
**Beneficial to gut microbiota**		
Avocados	Avocados	For each component, a score of 1 if consumption at or above the sex-specific median, else 0
Broccoli	Broccoli	
Chickpea	Chickpeas	
Coffee	Coffee	
Cranberries	Cranberries	
Fermented dairy	Yogurt, cheese, kefir, sour cream, buttermilk	
Fiber	Not applicable	
Green tea	Green tea	
Soybean	Soy products—Soy milk, Tofu	
Whole grains	Grains defined as whole grains, containing the entire grain kernel―the bran, germ, and endosperm	
**Unfavorable to gut microbiota**		
High-fat diet (% energy)	Not applicable	0 if consumption at or above 40% energy from fat, else 1 For each remaining component, a score of 0 if consumption at or above the sex-specific median, else 1
Processed meat	Frankfurters, sausages, corned beef, and luncheon meat that are made from beef, pork, or poultry	
Red meat	Beef, veal, pork, lamb, and game meat; excludes organ meat and cured meat	
Refined grains	Refined grains that do not contain all of the components of the entire grain kernel	

**Table 3:** Sample characteristics of study population, NHANES 2005–2010.

Characteristics	Sample for Correlation Analyses (*n* = 11,982)	Sample for Biomarker Analyses (*n* = 3812) ^a^
	Mean (SE) or *n* (%)	Mean (SE) or *n* (%)
Age in years	47.3 (0.36)	47.3 (0.48)
Sex		
Females	6177 (51.8)	1971 (51.8)
Males	5805 (48.2)	1841 (48.2)
Race and ethnicity		
Non-Hispanic White	6052 (72.1)	1891 (71.4)
Non-Hispanic Black	2302 (10.4)	744 (10.8)
Hispanic	3163 (12.2)	1026 (12.5)
Other including multi-racial	465 (5.3)	151 (5.5)
Marital status		
Married	6604 (59.1)	2133 (60.3)
Widowed, divorced or separated	2605 (17.9)	826 (18.1)
Living with partner	907 (7.2)	285 (6.9)
Never married	1866 (15.8)	568 (14.6)
Educational status		
Less than 12th grade	3269 (17.6)	1021 (17.5)
High school diploma	2884 (24.5)	934 (25.2)
Some college education	3337 (30.4)	1095 (31.9)
College graduate or above	2492 (27.5)	762 (25.4)
Smoking status		
Never smoked	6375 (53.6)	2042 (53.1)
Occasional smoker	423 (3.2)	116 (2.9)
Previous smoker	3111 (25.4)	990 (25.7)
Regular smoker	2073 (17.9)	664 (18.3)
Alcohol Use in the past 12 months		
None	3471 (24.7)	1083 (24.4)
12 drinks or more	8511 (75.3)	2729 (75.6)
Body mass index, kg/m^2^	28.7 (0.11)	28.7 (0.15)
Enterodiol (μg/g) ^b^	NA	1.53 (0.12)
Enterolactone (μg/g) ^b^	NA	9.04 (0.58)
DI-GM (ranges 0 to 13)	4.80 (0.04)	4.78 (0.04)
HEI-2015 (ranges 0 to 100)	53.5 (0.3)	53.3 (0.4)
Scaled HEI-2015 (ranges 0 to 10)	5.35 (0.03)	5.33 (0.04)
MDS (ranges 0 to 9)	3.80 (0.03)	3.79 (0.05)

*n* is unweighted sample size, ±SE—standard error, % percentage, mean values and percentages are weighted, NA—not available for the full sample, DI-GM—dietary index for gut microbiota, HEI-2015—healthy eating index, MDS—Mediterranean diet score. ^a^ Based on a subsample that includes no missing on enterodiol and enterolactone (*n* = 3812). ^b^ Levels are creatinine adjusted.

**Table 4:** Correlation between DI-GM and existing dietary indices, NHANES (*n* = 11,982).

	Correlation with DI-GM r (*p*-Value)
HEI-2015	0.54 (<0.0001)
MDS	0.42 (<0.0001)
Correlation between HEI-2015 and MDS	0.62 (<0.0001)

r—correlation coefficient, DI-GM—dietary index for gut microbiota, HEI-2015—healthy eating index, MDS—Mediterranean diet score.

**Table 5:** Correlation between creatinine-adjusted enterolignans and DI-GM components, DI-GM, HEI-2015, and MDS; NHANES (*n* = 3812).

	Enterodiol (μg/g) r (*p*-Value)	Enterolactone (μg/g) r (*p*-Value)
DI-GM	0.19 (<0.0001)	0.22 (<0.0001)
HEI-2015 scaled	0.23 (<0.0001)	0.25 (<0.0001)
MDS	0.16 (<0.0001)	0.19 (<0.0001)
Components of DI-GM		
Avocados	0.05 (0.01)	0.05 (0.007)
Broccoli	0.11 (<0.0001)	0.11 (<0.0001)
Chickpea	0.08 (0.01)	0.09 (0.002)
Coffee	0.01 (0.66)	0.09 (0.0007)
Cranberries	0.03 (0.15)	−0.01 (0.77)
Fermented dairy	−0.06 (0.01)	−0.06 (0.002)
Fiber	0.11 (<0.0001)	0.12 (<0.0001)
Soybean	0.13 (<0.0001)	0.08 (0.01)
Whole grains	0.09 (0.01)	0.11 (0.0003)
High-fat diet (>40% energy)	−0.02 (0.26)	−0.01 (0.82)
Processed meat	−0.08 (0.001)	−0.08 (<0.0001)
Red meat	−0.05 (0.01)	−0.11 (<0.0001)
Refined grains	−0.09 (0.0002)	−0.10 (<0.0001)

r—correlation coefficient, DI-GM—dietary index for gut microbiota, HEI-2015—healthy eating index—scaled to 0–10, MDS—Mediterranean diet score, enterodiol and enterolactone values were adjusted for creatinine and log-transformed.

**Table 6:** Association between DI-GM, existing dietary indices and creatinine-adjusted enterolignans, NHANES (*n* = 3812).

	Bivariate Association β [95%CI] (*p*-Value)			Adjusted Association ^a^ β [95% CI] (*p*-Value)		
Enterolignans	DI-GM 0–13	HEI-2015 0–10	MDS 0–9	DI-GM 0–13	HEI-2015 0–10	MDS 0–9
Enterodiol (μg/g)	0.19 [0.15, 0.23] (*p* < 0.0001)	0.29 [0.25, 0.34] (*p* < 0.0001)	0.16 [0.12, 0.20] (*p* < 0.0001)	0.12 [0.08, 0.17] (*p* < 0.0001)	0.21 [0.16, 0.26] (*p* < 0.0001)	0.11 [0.07, 0.15] (*p* < 0.0001)
Enterolactone (μg/g)	0.23 [0.18, 0.28] (*p* < 0.0001)	0.34 [0.28, 0.40] (*p* < 0.0001)	0.21 [0.16, 0.25] (*p* < 0.0001)	0.14 [0.09, 0.18] (*p* < 0.0001)	0.20 [0.15, 0.26] (*p* < 0.0001)	0.12 [0.08, 0.17] (*p* < 0.0001)

β [95% CI]—regression coefficient [95% confidence interval], DI-GM—dietary index for gut microbiota, HEI-2015—healthy eating index—scaled to 0–10, MDS—Mediterranean diet score, enterodiol and enterolactone values were adjusted for creatinine and log-transformed. ^a^ adjusted for age, sex, race, marital status, education, smoking, alcohol use, and BMI.

## Data Availability

Data described in the manuscript, code book, and analytic code will be made available upon reasonable request of the corresponding author.

## Supplementary Materials

The following supporting information can be downloaded at: https://www.mdpi.com/article/10.3390/nu16071045/s1, Supplementary Material Section S1: Search Strings for Pubmed and Embase Databases; Supplementary Material Section S2: Gut Microbiota Outcomes; Table S1: Summary Tables Presenting Extracted Evidence from Reviewed Articles.
