# Supplementary material for: The Development and Evaluation of a Literature-Based Dietary Index for Gut Microbiota
Source: Nutrients. 2024 Apr 3;16(7):1045. doi: 10.3390/nu16071045 (PMC11013161; doi:10.3390/nu16071045)
Supplement: Supplementary file 1 [file nutrients-16-01045-s001.zip › nutrients-2875045-supplementary.pdf]

Supplementary Material S1: Search Strings for Pubmed and Embase Databases

1. PubMed (n=5,828)

(((("gastrointestinal tract"[MeSH Terms] OR "gastrointestin*"[Title/Abstract] OR "Gut"[Title/Abstract] OR "intestin*"[Title/Abstract] OR "colon*"[Title/Abstract] OR "feces"[MeSH Terms] OR "feces"[Title/Abstract] OR "faecal"[Title/Abstract]) AND ("microbiota"[MeSH Terms] OR "gastrointestinal microbiome"[MeSH Terms] OR "dysbiosis"[MeSH Terms] OR ("alpha diversity"[Title/Abstract] OR "alpha diversity"[Title/Abstract] OR "Acetate"[Title/Abstract] OR "Bacteroides"[Title/Abstract] OR "Bacteroidetes"[Title/Abstract] OR "beta diversity"[Title/Abstract] OR "beta diversity"[Title/Abstract] OR "Bifidobacterium"[Title/Abstract] OR "Butyrate"[Title/Abstract]) OR ("bacteria"[Title/Abstract] OR "flora"[Title/Abstract] OR "Microbial"[Title/Abstract] OR "microflora"[Title/Abstract] OR "dysbiosis"[Title/Abstract] OR "microbial profile"[Title/Abstract] OR "Firmicutes"[Title/Abstract] OR "microbial communit*"[Title/Abstract] OR "microbes"[Title/Abstract] OR "metabolom*"[Title/Abstract] OR "microbial composition"[Title/Abstract] OR "microbial diversity"[Title/Abstract] OR "microbiome"[Title/Abstract] OR "microbiota"[Title/Abstract] OR "shannon diversity index"[Title/Abstract] OR "shannon index"[Title/Abstract] OR ("short chain fatty acid*"[Title/Abstract] OR "scfa"[Title/Abstract]))) AND ("diet"[MeSH Terms] OR "food"[MeSH Terms] OR "nutrients"[MeSH Terms] OR "diet*"[Title/Abstract] OR "energy intake*"[Title/Abstract] OR "food*"[Title/Abstract] OR "macronutrient*"[Title/Abstract] OR "nutrient*"[Title/Abstract] OR ("adzuki"[Title/Abstract] OR "bean*"[Title/Abstract] OR "cannellini"[Title/Abstract] OR "chickpea*"[Title/Abstract] OR "dietary pulse*"[Title/Abstract] OR "fava"[Title/Abstract] OR "faba"[Title/Abstract] OR "haricots"[Title/Abstract] OR

"legum*"[Title/Abstract] OR "lentil*"[Title/Abstract] OR "lupin*"[Title/Abstract] OR "lupinus"[Title/Abstract] OR "mung"[Title/Abstract] OR "Pea"[Title/Abstract] OR "Peas"[Title/Abstract]) OR ("Nuts"[MeSH Terms] OR "almond*"[Title/Abstract] OR "cashew*"[Title/Abstract] OR "chestnut*"[Title/Abstract] OR "groundnut*"[Title/Abstract] OR "hazelnut*"[Title/Abstract] OR "macadamia*"[Title/Abstract] OR "Nut"[Title/Abstract] OR "Nuts"[Title/Abstract] OR "peanut*"[Title/Abstract] OR "pecan*"[Title/Abstract] OR "pistachio*"[Title/Abstract] OR "walnut*"[Title/Abstract]) OR ("soy foods"[MeSH Terms] OR "plant proteins"[MeSH Terms] OR "Miso"[Title/Abstract] OR "soybean*"[Title/Abstract] OR "Soy"[Title/Abstract] OR "Tofu"[Title/Abstract]) OR ("grain proteins"[MeSH Terms] OR "whole grains"[MeSH Terms] OR "amaranth"[Title/Abstract] OR "barley"[Title/Abstract] OR "bran"[Title/Abstract] OR "bread"[Title/Abstract] OR "buckwheat"[Title/Abstract] OR "bulgar"[Title/Abstract] OR "canary seed*"[Title/Abstract] OR "cereal*"[Title/Abstract] OR "corn"[Title/Abstract] OR "couscous"[Title/Abstract] OR "durum"[Title/Abstract] OR "einkorn"[Title/Abstract] OR "emmer"[Title/Abstract] OR "farro"[Title/Abstract] OR "grain*"[Title/Abstract] OR "kamut"[Title/Abstract] OR "maize"[Title/Abstract] OR "millet"[Title/Abstract] OR "oats"[Title/Abstract] OR "pasta"[Title/Abstract] OR "quinoa"[Title/Abstract] OR "rice"[Title/Abstract] OR "rye"[Title/Abstract] OR "spelta"[Title/Abstract] OR "spelt"[Title/Abstract] OR "sorghum"[Title/Abstract] OR "teff"[Title/Abstract] OR "triticale"[Title/Abstract] OR "whole grain*"[Title/Abstract] OR "wholegrain*"[Title/Abstract] OR "wheat"[Title/Abstract]) OR ("dairy products"[MeSH Terms] OR "milk proteins"[MeSH Terms] OR "butter"[Title/Abstract] OR "buttermilk"[Title/Abstract] OR "casein"[Title/Abstract] OR "cheese"[Title/Abstract] OR "cream"[Title/Abstract] OR "dair*"[Title/Abstract] OR "ghee"[Title/Abstract] OR "kefir"[Title/Abstract] OR

"milk"[Title/Abstract] OR "whey"[Title/Abstract] OR "yogurt"[Title/Abstract] OR "yoghurt"[Title/Abstract]) OR ("egg proteins, dietary"[MeSH Terms] OR "fish proteins, dietary"[MeSH Terms] OR "meat"[MeSH Terms] OR "bacon*"[Title/Abstract] OR "beef"[Title/Abstract] OR "chicken"[Title/Abstract] OR "duck"[Title/Abstract] OR "Egg"[Title/Abstract] OR "Eggs"[Title/Abstract] OR "Fish"[Title/Abstract] OR "goat"[Title/Abstract] OR "ham"[Title/Abstract] OR "hot dog*"[Title/Abstract] OR "lamb"[Title/Abstract] OR "liver"[Title/Abstract] OR "meat*"[Title/Abstract] OR "mutton"[Title/Abstract] OR "pastrami"[Title/Abstract] OR "pork"[Title/Abstract] OR "poultry"[Title/Abstract] OR "rabbit"[Title/Abstract] OR "salami*"[Title/Abstract] OR "sausage*"[Title/Abstract] OR "seafood"[Title/Abstract] OR "turkey"[Title/Abstract] OR "veal"[Title/Abstract]) OR ("fruit"[MeSH Terms] OR "fruit proteins"[MeSH Terms] OR "apple*"[Title/Abstract] OR "apricot*"[Title/Abstract] OR "avocado*"[Title/Abstract] OR "banana*"[Title/Abstract] OR "berries"[Title/Abstract] OR "blackberr*"[Title/Abstract] OR "blueberr*"[Title/Abstract] OR "cantaloupe"[Title/Abstract] OR "cherr*"[Title/Abstract] OR "citrus"[Title/Abstract] OR "cranberr*"[Title/Abstract] OR "fruit*"[Title/Abstract] OR "grape*"[Title/Abstract] OR "grapefruit*"[Title/Abstract] OR "guava*"[Title/Abstract] OR "juice*"[Title/Abstract] OR "kiwi*"[Title/Abstract] OR "lingonberr*"[Title/Abstract] OR "mandarin*"[Title/Abstract] OR "mango*"[Title/Abstract] OR "melon*"[Title/Abstract] OR "nectarine*"[Title/Abstract] OR "orange*"[Title/Abstract] OR "papaya*"[Title/Abstract] OR "peach*"[Title/Abstract] OR "pear"[Title/Abstract] OR "pears"[Title/Abstract] OR "pineapple*"[Title/Abstract] OR "plum"[Title/Abstract] OR "plums"[Title/Abstract] OR "prune*"[Title/Abstract] OR "raspberr*"[Title/Abstract] OR "raisin"[Title/Abstract] OR "raisins"[Title/Abstract] OR "satsuma*"[Title/Abstract] OR "strawberr*"[Title/Abstract] OR

"tangerine*"[Title/Abstract] OR "berry"[Title/Abstract]) OR ("vegetables"[MeSH Terms] OR "artichoke*"[Title/Abstract] OR "asparagus"[Title/Abstract] OR "aubergine*"[Title/Abstract] OR "beetroot*"[Title/Abstract] OR "beets"[Title/Abstract] OR "broccoli*"[Title/Abstract] OR "brussel sprout*"[Title/Abstract] OR "cabbage*"[Title/Abstract] OR "carrot*"[Title/Abstract] OR "cauliflower*"[Title/Abstract] OR "celery"[Title/Abstract] OR "chard"[Title/Abstract] OR "cucumber*"[Title/Abstract] OR "fennel"[Title/Abstract] OR "garlic"[Title/Abstract] OR "kale"[Title/Abstract] OR "leek*"[Title/Abstract] OR "lettuce*"[Title/Abstract] OR "okra"[Title/Abstract] OR "onion*"[Title/Abstract] OR "parsnip*"[Title/Abstract] OR "pepper*"[Title/Abstract] OR "potato*"[Title/Abstract] OR "pumpkin*"[Title/Abstract] OR "rhubarb"[Title/Abstract] OR "salad*"[Title/Abstract] OR "spinach*"[Title/Abstract] OR "squash"[Title/Abstract] OR "tomato*"[Title/Abstract] OR "turnip*"[Title/Abstract] OR "vegetable*"[Title/Abstract] OR "yams"[Title/Abstract] OR "yam"[Title/Abstract]) OR ("dietary fats"[MeSH Terms] OR "fiber*"[Title/Abstract] OR "fibre*"[Title/Abstract] OR "vitamin c"[Title/Abstract] OR "vitamin e"[Title/Abstract] OR "ascorbic acid"[Title/Abstract] OR "carotenoid*"[Title/Abstract] OR "flavonoid*"[Title/Abstract] OR "beta glucan*"[Title/Abstract] OR "oil"[Title/Abstract] OR "oils"[Title/Abstract] OR "margarine"[Title/Abstract] OR "polyphenol*"[Title/Abstract]) OR ("beverages"[MeSH Terms] OR "beverages"[MeSH Terms] OR "beverage*"[Title/Abstract] OR "caffein*"[Title/Abstract] OR "candy"[Title/Abstract] OR "candies"[Title/Abstract] OR "chocolate*"[Title/Abstract] OR "coffee"[Title/Abstract] OR "drink"[Title/Abstract] OR "honey"[Title/Abstract] OR "juice*"[Title/Abstract] OR "pop"[Title/Abstract] OR "salt"[Title/Abstract] OR "soda"[Title/Abstract] OR "sodium"[Title/Abstract] OR "sugar*"[Title/Abstract] OR "sweet*"[Title/Abstract] OR "tea"[Title/Abstract] OR "dietary sugars"[MeSH Terms]))) NOT

("animals"[MeSH Terms] NOT "humans"[MeSH Terms])) AND ("cohort studies"[MeSH Terms:noexp] OR "longitudinal studies"[MeSH Terms:noexp] OR "follow up studies"[MeSH Terms:noexp] OR "prospective studies"[MeSH Terms:noexp] OR "retrospective studies"[MeSH Terms:noexp] OR "cohort"[Title/Abstract] OR "longitudinal"[Title/Abstract] OR "prospective"[Title/Abstract] OR "retrospective"[Title/Abstract] OR ("Clinical Trial"[Publication Type:noexp] OR "clinical trial, phase i"[Publication Type] OR "clinical trial, phase ii"[Publication Type] OR "clinical trial, phase iii"[Publication Type] OR "clinical trial, phase iv"[Publication Type] OR "controlled clinical trial"[Publication Type] OR "multicenter study"[Publication Type] OR "randomized controlled trial"[Publication Type] OR "Clinical Trials as Topic"[MeSH Terms:noexp] OR "clinical trials, phase i as topic"[MeSH Terms:noexp] OR "clinical trials, phase ii as topic"[MeSH Terms:noexp] OR "clinical trials, phase iii as topic"[MeSH Terms:noexp] OR "clinical trials, phase iv as topic"[MeSH Terms:noexp] OR "controlled clinical trials as topic"[MeSH Terms:noexp] OR "randomized controlled trials as topic"[MeSH Terms:noexp] OR "early termination of clinical trials"[MeSH Terms:noexp] OR "multicenter studies as topic"[MeSH Terms:noexp] OR "Double-Blind Method"[MeSH Terms] OR (("randomised"[Title/Abstract] OR "randomized"[Title/Abstract]) AND ("trial"[Title/Abstract] OR "trials"[Title/Abstract])) OR (("single"[Title/Abstract] OR "double"[Title/Abstract] OR "doubled"[Title/Abstract] OR "triple"[Title/Abstract] OR "tripled"[Title/Abstract] OR "treble"[Title/Abstract] OR "treble"[Title/Abstract]) AND ("blind*"[Title/Abstract] OR "mask*"[Title/Abstract])) OR ("4 arm"[Title/Abstract] OR "four arm"[Title/Abstract]) OR "evaluation studies"[Publication Type] OR "evaluation studies as topic"[MeSH Terms:noexp] OR "program evaluation"[MeSH Terms:noexp] OR "validation studies as topic"[MeSH Terms:noexp] OR ("pre"[Title/Abstract] AND "post"[Title/Abstract])

OR ("pretest"[Title/Abstract] AND "posttest"[Title/Abstract]) OR ("program*"[Title/Abstract] AND ("evaluat*"[Title/Abstract] OR "effectiveness"[Title/Abstract])) OR "intervention"[Title/Abstract]))) AND ((english[Filter]) AND (2008:2021[pdat]))

2. Embase (n=9,695)

('crossover procedure':de OR 'double-blind procedure':de OR 'randomized controlled trial':de OR 'single-blind procedure':de OR random*:de,ab,ti OR factorial*:de,ab,ti OR crossover*:de,ab,ti OR ((cross NEXT/1 over*):de,ab,ti) OR placebo*:de,ab,ti OR ((doubl* NEAR/1 blind*):de,ab,ti) OR ((singl* NEAR/1 blind*):de,ab,ti) OR assign*:de,ab,ti OR allocat*:de,ab,ti OR volunteer*:de,ab,ti OR 'controlled clinical trial'/exp OR trial:ti,ab OR 'clinical article'/exp OR 'controlled study'/exp OR 'major clinical study'/exp OR 'prospective study'/exp OR 'cohort analysis'/exp OR 'cohort':ti,ab OR 'compared':ti,ab OR 'groups':ti,ab OR 'multivariate':ti,ab OR 'program evaluation'/exp OR 'follow-up studies'/exp OR 'cross-over studies'/exp OR 'pretest posttest control group design'/exp OR 'control group'/exp OR 'parallel design'/exp) AND ('gastrointestinal tract'/exp OR 'gastrointestinal tract' OR gastrointestin*:ab,ti OR gut:ab,ti OR 'intestine'/exp OR 'intestine' OR intestin*:ab,ti OR colon:ab,ti OR 'feces'/exp OR 'feces' OR feces:ab,ti OR faecal:ab,ti OR colon*:ti,ab) AND ('intestine flora'/exp OR 'feces microflora'/exp OR 'dysbiosis'/exp OR 'bacterial microbiome'/exp OR 'α diversity':ab,ti OR 'alpha diversity':ab,ti OR acetate:ab,ti OR bacteroides:ab,ti OR bacteroidetes:ab,ti OR 'β diversity':ab,ti OR 'beta diversity':ab,ti OR bifidobacterium:ab,ti OR butyrate:ab,ti OR bacteria:ab,ti OR flora:ab,ti OR microbial:ab,ti OR microflora:ab,ti OR dysbiosis:ab,ti OR 'microbial profile':ab,ti OR firmicutes:ab,ti OR 'microbial communit*':ab,ti OR microbes:ab,ti OR metabolom*:ab,ti OR 'microbial composition':ab,ti OR 'microbial diversity':ab,ti OR microbiome:ab,ti OR microbiota:ab,ti OR 'shannon diversity index':ab,ti OR 'shannon index':ab,ti OR 'short chain fatty

acid*':ab,ti OR scfa:ab,ti OR 'microflora'/exp) AND ('diet'/exp OR 'dietary intake'/exp OR 'food'/exp OR 'nutrient'/exp OR food*:ab,ti OR diet*:ab,ti OR 'energy intake*':ab,ti OR macronutrient*:ab,ti OR nutrient*:ab,ti OR 'soybean'/exp OR 'soybean protein'/exp OR 'soy food'/exp OR tofu:ab,ti OR miso:ab,ti OR soy:ab,ti OR soybean*:ab,ti OR 'food grain'/exp OR 'whole grain*':ab,ti OR amaranth:ab,ti OR barley:ab,ti OR bran:ab,ti OR bread:ab,ti OR buckwheat:ab,ti OR bulgar:ab,ti OR 'canary seed*':ab,ti OR cereal*:ab,ti OR corn:ab,ti OR couscous:ab,ti OR 'triticum durum':ab,ti OR einkorn:ab,ti OR emmer:ab,ti OR farro:ab,ti OR grain*:ab,ti OR kamut:ab,ti OR maize:ab,ti OR millet:ab,ti OR oat:ab,ti OR oats:ab,ti OR pasta:ab,ti OR quinoa:ab,ti OR rice:ab,ti OR rye:ab,ti OR spelt:ab,ti OR spelta:ab,ti OR sorghum:ab,ti OR teff:ab,ti OR triticale:ab,ti OR wheat:ab,ti OR 'nut'/exp OR almond*:ab,ti OR cashew*:ab,ti OR 'chestnut'/exp OR chestnut*:ab,ti OR groundnut*:ab,ti OR peanut*:ab,ti OR 'hazelnut'/exp OR hazelnut*:ab,ti OR macadamia*:ab,ti OR nut:ab,ti OR nuts:ab,ti OR pecan*:ab,ti OR pistachio*:ab,ti OR 'walnut'/exp OR walnut*:ab,ti OR 'legume'/exp OR adzuki:ab,ti OR bean*:ab,ti OR cannellini:ab,ti OR chickpea*:ab,ti OR 'dietary pulse*':ab,ti OR faba:ab,ti OR fava:ab,ti OR haricots:ab,ti OR legum*:ab,ti OR lentil*:ab,ti OR lupin*:ab,ti OR 'mung bean':ab,ti OR pea:ab,ti OR peas:ab,ti OR 'dairy products'/exp OR butter:ab,ti OR buttermilk:ab,ti OR casein:ab,ti OR cheese:ab,ti OR cream:ab,ti OR dair*:ab,ti OR ghee:ab,ti OR kefir:ab,ti OR milk:ab,ti OR whey:ab,ti OR yoghurt:ab,ti OR yogurt:ab,ti OR 'protein intake'/exp OR 'egg'/exp OR 'fish consumption'/exp OR 'meat'/exp OR bacon:ab,ti OR beef:ab,ti OR chicken:ab,ti OR duck:ab,ti OR egg:ab,ti OR eggs:ti,ab OR goat:ab,ti OR ham:ab,ti OR 'hot dog*':ab,ti OR lamb:ab,ti OR liver:ab,ti OR meat*:ab,ti OR mutton:ab,ti OR pastrami:ab,ti OR pork:ab,ti OR poultry:ab,ti OR rabbit:ab,ti OR salami*:ab,ti OR sausage*:ab,ti OR 'seafood*':ab,ti OR turkey:ab,ti OR veal:ab,ti OR 'fruit'/exp OR apple*:ab,ti OR apricot:ab,ti OR

avocado:ab,ti OR banana*:ab,ti OR berry:ab,ti OR berries:ab,ti OR blackberr*:ab,ti OR cantaloupe*:ab,ti OR cherry:ab,ti OR citrus:ab,ti OR cranberr*:ab,ti OR fruit*:ab,ti OR grape*:ab,ti OR grapefruit*:ab,ti OR guava*:ab,ti OR kiwi*:ab,ti OR lingonberr*:ab,ti OR mandarin*:ab,ti OR mango*:ab,ti OR melon*:ab,ti OR nectarine*:ab,ti OR orange*:ab,ti OR papaya*:ab,ti OR peach*:ab,ti OR pear:ab,ti OR pears:ab,ti OR pineapple*:ab,ti OR plum:ab,ti OR plums:ab,ti OR prune*:ab,ti OR raspberr*:ab,ti OR raisin*:ab,ti OR satsuma*:ab,ti OR tangerine*:ab,ti OR 'vegetable'/exp OR artichoke*:ab,ti OR asparagus:ab,ti OR aubergine*:ab,ti OR beetroot*:ab,ti OR beets:ab,ti OR broccoli*:ab,ti OR 'brussel sprout*':ab,ti OR cabbage*:ab,ti OR carrot*:ab,ti OR cauliflower*:ab,ti OR celery:ab,ti OR chard:ab,ti OR cucumber*:ab,ti OR fennel:ab,ti OR garlic:ab,ti OR kale:ab,ti OR leek*:ab,ti OR lettuce*:ab,ti OR okra:ab,ti OR onion*:ab,ti OR parsnip*:ab,ti OR pepper*:ab,ti OR potato*:ab,ti OR pumpkin*:ab,ti OR rhubarb:ab,ti OR salad*:ab,ti OR spinach*:ab,ti OR squash:ab,ti OR tomato*:ab,ti OR turnip*:ab,ti OR vegetable*:ab,ti OR yams:ab,ti OR yam:ab,ti OR 'fat intake'/exp OR fiber:ab,ti OR fibre*:ab,ti OR 'vitamin c':ab,ti OR 'vitamin e':ab,ti OR 'ascorbic acid':ab,ti OR carotenoid*:ab,ti OR flavonoid*:ab,ti OR 'beta glucan*':ab,ti OR oil:ab,ti OR oils:ab,ti OR margarine:ab,ti OR polyphenol*:ab,ti OR 'beverage'/exp OR beverage*:ab,ti OR caffein*:ab,ti OR candy:ab,ti OR candies:ab,ti OR chocolate*:ab,ti OR coffee:ab,ti OR drink:ab,ti OR honey:ab,ti OR juice*:ab,ti OR pop:ab,ti OR salt:ab,ti OR soda:ab,ti OR sodium:ab,ti OR sugar*:ab,ti OR sweet*:ab,ti OR tea:ab,ti OR 'sugar intake'/exp) NOT ([animals]/lim NOT [humans]/lim) AND [english]/lim AND [2008-2021]/py AND [embase]/lim NOT ([embase]/lim AND [medline]/lim)

Supplementary Material 2: Gut Microbiota Outcomes

- Change in alpha diversity (microbial diversity within each subject) and beta diversity (difference between each subject's) – Increase indicates beneficial effect, and the reverse indicates harmful effect
    - α-diversity indices (Observed species, Shannon index, Simpson index, and Chao 1 index)
    - β-diversity dissimilarity based on Bray-Curtis distance
- Change in fecal short chain fatty acid (SCFA) levels
    - Beneficial change - Increase in total SCFA, butyrate, acetate, propionate, and Isobutyrate
- Change at phyla level – change in abundance of phyla Bacteroidetes, Firmicutes, Proteobacteria, Actinobacteria, Verrucomicrobia, Tenericutes, Fusobacteria, Saccharilbacteria, Chloroflexi, Acidobacteria, and Cyanobacteria
    - Beneficial phyla
        - Bacteroidetes (gram-negative bacteria - Bacteroides, Prevotella, Porphyromonas, Alistipes and Parabacteroides)
        - Firmicutes (gram-positive bacteria - butyrate producers – Lactobacillus (pro), Faecalibacterium (pro), Eubacterium, Roseburia, Anaerostipes, Clostridium perfringens and Ruminococcus)
        - Actinobacteria (Bifidobacteria)
    - Not beneficial when excess phyla
        - Proteobacteria (Enterobacteriaceae family, Escherichia genus, Collinsella genus)
        - Fusobacteria - Fusobacterium nucleatum

- Change in ratios of phyla – change in Firmicutes/Bacteroidetes ratio
    - Balanced Firmicutes/Bacteroidetes ratio – Beneficial
    - Increase in Firmicutes/Bacteroidetes ratio – associated with obesity
- Change in bacteria that are shown to be associated with inflammation, obesity, postprandial glucose metabolism, cardiometabolic health
    - Beneficial bacteria - Prevotella copri , Blastocystis spp., Faecalibacterium prausnitzii, Haemophilus parainfluenzae , Firmicutes bacterium CAG95, Eubacterium. eligens, Roseburia CAG182, Oscillibacter sp 57_20, Firmicutes bacterium CAG170, Oscillibacter sp PC13, Clostridium sp CAG167, Bifidobacterium. animalis, Romboutsia ilealis, Veillonella atypica, V. infantium, V. dispar
    - Harmful bacteria - Clostridia (C. spiroforme, C.bolteae CAG59, C. bolteae, Clostridium CAG58, C. symbiosum, C. innocuum, C. leptum), Flavonifractor plautii, Rumminococcus. gnavus, Eggerthella lenta, E coli, Ruthenibacterium. lactatiformans, Collinsella intestinalis, Blautia hydrogenotrophica, Anaerotruncus colihominis, Bacteroides thetaiotaomicron
- Change in bacteria that are shown to be associated with different cancers (colorectal cancer, breast cancer, other cancers) Beneficial bacteria - Bifidobacterium, Lactobacillus, Lactococcus, Roseburia, Akkermansia, Faecalibacterium prausnitzii, Eubacterium rectale, Roseburia faecis, Eulonchus halli, Prevotella copri, Parabacteroides distasonis, Sphingomonadaceae
- Harmful bacteria - Bacteroides fragilis, Enterobacteriaceae spp., Listeria monocytogenes, Fusobacterium nucleatum, Streptococcus gallolyticus, Clostridium difficile, Clostridium

septicum, Enterococcus faecalis, Escherichia coli, Peptostreptococcus stomatis, Helicobacter pylori, Ruminococcus torques, Bilophila wadsworthia, Streptococcus agalactiae, Pseudomonas, Alistipes, Atopobium, Hydrogenophaga, Gluconacetobacter, Bacillus cereus.

- Most reported bacteria from the studies included in the review
- Beneficial - Faecalibacterium, Bifidobacterium, Lactobacillus, Lactococcus, Parabacteroides, Roseburia, Eubacterium rectale, Eubacterium hallii, Akkermansia, Akkermansia muciniphila, Prevotella, Prevotella copri, Anaerostipes, Anaerostipes hadrus, Veillonellaceae, Parabacteroides distasonis, Gemmiger, Moraxellaceae
- Harmful - Bacteroides, Bacteroides fragilis, Fusobacteria, Streptococcus, Clostridium, Clostridium symbiosum, Clostridium perfringens, Dialister, Alistipes, Bilophila, Ruminococcus gnavus, Dorea, Actinomyces, Odoribacter, Blautia, Lachnospira, Lachnospiraceae, Sutterella, Enterobacteriaceae, Klebsiella sp
- Not clearly known - Ruminococcus bromi, Oscillospira

Table S1: Summary Tables Presenting Extracted Evidence from Reviewed Articles

Table S1.a. Summary tables – Dairy

**Specific foods -** Dairy (n=8)	**No of studies**	**Type of design**	**Beneficial effect**	**Harmful effect**	**No effect**
Kefir - 180 mL/day - Bellikci-Koyu_2019	1	Randomized, Controlled Study	Increase in the Actinobacteria		No effect on other phyla or diversity
High dairy (HD: 1500 mg calcium/day) versus low dairy (LD: 600 mg calcium/day). -Bendtsen_2018<br><br>(HD, consumed ≥4 servings of dairy/day vs AD ≤2/day) - Khorraminezhad_2021<br><br>High-dairy diet (HDD) (5–6 dairy portions per day) and a low-dairy diet (LDD) (≤1 dairy portion - Swarte 2020	3	2 x A randomized controlled parallel design<br><br>A randomized, crossover Study	The abundance of Lactococcus were significantly increased during the HDD, while the relative abundance of Bilophila significantly decreased.<br>Increase in Faecalibacterium after HD	The abundance of Streptococcus significantly increased during the HDD	No significant changes from the phylum to the genus level or in diversity<br><br>Ratio Bacteroidetes /firmicutes showed no differences.
Total dairy intake - highest (≥0.5 serving/d) and second highest (1 serving/wk-0.5 serving/d) total dairy intake category, compared with lowest category (<1 serving/mo - Shuai_2021	1	Prospective cohort study	High dairy intake - higher levels of Shannon, Simpson index and beta-diversity.<br>Yogurt consumption was positively associated with Shannon and Observed species.<br>Bifidobacterium were enriched in in the highest total dairy intake group	Streptococcus, and Clostridium were enriched in in the highest total dairy intake group	
Whole-fat liquid yogurt - 220 g/day - Chen_2019<br><br>Three types of yogurts (cow's whole-milk yogurt, ewe's semi-skimmed-milk yogurt, ewe's whole-milk yogurt) - Redondo_2019	2	A randomized, controlled trial	Decreased the relative abundance of the Firmicutes phylum		No differential effect by type of yogurt

**Specific foods -** Dairy (n=8)	**No of studies**	**Type of design**	**Beneficial effect**	**Harmful effect**	**No effect**
Whole milk – 250ml/day - Li_2018	1	Intervention study			No effect on diversity or on SCFA

Table S1.b. Summary tables – Meat and fish products

**Specific foods -** Meat and fish products (n=7)	**No of studies**	**Type of design**	**Beneficial effect**	**Harmful effect**	**No effect**
A standard diet enriched with 100 g of sardines 5 d/wk - Balfegó_2016	1	A pilot randomized trial	A decrease in Firmicutes/Bacteroidetes ratio<br>An increase in Bacteroides–Prevotella	1	A pilot randomized trial
Atlantic cod in weekly doses of 750 g, Atlantic salmon in weekly doses of 750 g, and no-fish intake - Bratlie_2021	1	A randomized, controlled parallel group design	Higher counts for a few bacteria in the Firmicutes phylum	In the Cod and Salmon groups - lower counts for bacteria that belong to the Bacteroidales order in the Bacteroidetes phylum and the Clostridiales order of the Firmicutes phyla	
Fried meat - 4 times/week vs no fried meat intake - Gao_2021	1	Randomized controlled-feeding trial	The ratio of Firmicutes and Bacteroidetes was decreased in both groups, and it was significantly lower in the control group		No difference in microbial community richness
Consume 2x150 g portions of farmed salmon per week or habitual diet - Urwin_2014	1	A single-blind, randomized, controlled intervention			No significant effects on any of the bacteria enumerated in maternal fecal samples
High-protein/high red meat (HP) diet (35% protein, 40% carbohydrate, and 25% fat) VS High-carbohydrate/low red meat (HC) diet (17% protein, 58% carbohydrate, and 25% fat) - Benassi-Evans_2010	1	A Parallel-Group, Randomized, Controlled Study			No change in SCFA

**Specific foods -** Meat and fish products (n=7)	**No of studies**	**Type of design**	**Beneficial effect**	**Harmful effect**	**No effect**
'Animal-based diet'- composed of meats, eggs, and cheeses VS 'Plant-based diet'- rich in grains, legumes, fruits, and vegetables - David_2014<br><br>Mediterranean diet - including meat and meat products, poultry, and fish VS Vegetarian diet - abstinence to consume meat and meat products, poultry, fish, seafood and flesh from any other animal, but included eggs and dairy products - Pagliai_2020	2	Dietary intervention<br><br>Dietary intervention study	Decrease in the levels of Firmicutes that metabolize dietary plant polysaccharides (Roseburia, Eubacterium rectale and Ruminococcus bromii) in animal-based diet<br><br>MD led to an increase of 10% in propionate<br>Carbohydrate was positively correlated with butyric acid in MD	Reduction in fecal propionate and an increase in both branched chain SCFA, isobutyric and isovaleric acid after vegetarian diet<br>Increase in the abundance of bile-tolerant microorganisms (Alistipes, Bilophila and Bacteroides) after animal-based diet<br>Fat intake was negatively correlated with acetic acid, propionic acid, valeric acid and isobutyric acid	No change in alpha diversity<br>No change in alpha diversity

Table S1.c. Summary tables – Legumes and nuts

**Specific foods- Legumes_nuts** (n=15)	**No of studies**	**Type of design**	**Beneficial effect**	**Harmful effect**	**No effect**
Daily intake of flaxseed mucilage (10 g) or placebo for 6 wks - Brahe_2015<br><br>Flax Seed ground - 10 g/d of for 6 weeks - McCann_2021 - McCann_2021	2	RCT, a single-blinded, parallel-group<br><br>A randomized, crossover intervention study	Fusobacteria, Pyramidobacter and Odoribacter -reduced in the FS	alpha diversity- Shannon diversity index and Simpson's inverse index decreased<br>Clostridium genus – increased<br>Faecalibacterium prausnitzii species & Ruminococcuslactaris - decreased	Did not differ in Alpha and beta diversity (Shannon Diversity Index)
Almonds -1.5oz daily for 3wks - Burns 2016<br><br>Almonds - 56g/day for 8wks - Choo 2021<br><br>Almond - 57 g/d for 8 wk - Dhillon_2019<br><br>Almonds – 42 g/day for 3wks - Holscher_2018_2<br><br>Almond-based low carbohydrate diet (a-LCD) - 56 g/day almonds vs a low-fat diet (LFD) - Ren_2020<br><br>No nuts vs 1.5 servings/d either almonds or pistachios (42.5 g/d) vs 3 servings/d of	6	3x A randomized, crossover study<br><br>A randomized, parallel controlled trial<br><br>2xA randomized, controlled, parallel arm	Increases in bacterial community richness, evenness (Simpson's index(1−D), P=.003) and diversity (Faith's phylogenetic diversity<br><br>Increase in alpha diversity & beta-diversity<br><br>Decrease in Bacteroides fragilis<br>Increased Lachnospira, Roseburia, Clostridium, and Dialister<br><br>Increase in Roseburia, Ruminococcus, and Eubacterium in the a-LCD arm<br><br>β-diversity larger for pistachio	Increases in the relative abundance of OTUs assigned to three members in the Ruminococcaceae family and one member of the Lachnospiraceae family<br><br>Decreased Actinobacteria, Bifidobacterium and Parabacteroides<br><br>Decrease in Firmicutes in the a-LCD arm<br>Decrease in Bacteroidetes and Bacteroides in the a-LCD arm<br><br>Decrease in lactic acid bacteria after 42.5 or 85 g/d of pistachios	No significant differences in alpha diversity<br><br>No significant change at phylum level<br><br>No change in SCFA<br><br>α-diversity was not affected by either almonds or pistachios<br><br>No change at phyla level

**Specific foods- Legumes_nuts** (n=15)	**No of studies**	**Type of design**	**Beneficial effect**	**Harmful effect**	**No effect**
either almonds or pistachios (85 g/d) - Ukhanova_2014					
Canned Chickpea - 200 g/d for 3wks - Fernando_2010	1	A randomized crossover intervention	Increase in Faecalibacterium prausnitzii<br>A decrease in Clostridium clusters (C. histolyticum, C. lituseburense and relatives)		No change in alpha diversity, SCFA
Walnut - 1.5 servings (42g)/day for 3 wks - Holscher_2018<br><br>Whole walnuts (WD; 57–99 g/d walnuts; 2.7% α-linolenic acid (ALA)), vs a fatty acid–matched diet devoid of walnuts (walnut fatty acid–matched diet; WFMD; 2.6% ALA), vs a diet replacing ALA with oleic acid without walnuts - Tindall_2020	2	A controlled-feeding, randomized crossover study	Change in beta-diversity<br>Increased Faecalibacterium, and Roseburia<br><br>Decreased Ruminococcus, Dorea, and Oscillospira<br><br>Increase in Roseburia, Eubacterium eligens group in WD arm	Increased Firmicutes and decreased Actinobacteria<br><br>Increase Clostridium, Dialister<br><br>Decrease in Bifidobacterium<br><br>Increase in Lachnospiraceae, Gordonibacter in WD	No change in alpha diversity<br>No change in alpha diversity<br>No change in Bacteroidetes, Firmicutes, or Proteobacteria
Non-fermented soybean milk (NFSM - 100 g/day) or fermented soybean milk (FSM-100 g/day) for 2 weeks - Inoguchi_2012<br><br>One Revival soy bar per day (160 mg of soy isoflavones and 1 g saponin) - Nakatsu_2014<br><br>Low glycinin soymilk - 500 mL/day(LGS, 49.5% β-conglycinin/6% glycinin) VS conventional soymilk (S, 26.5% β-conglycinin/38.7% glycinin) VS bovine milk (M, 0% β-conglycinin/0% glycinin) - Fernandez-Raudales_2012	3	Randomized controlled-feeding trial<br><br>Experimental design<br><br>Randomized, double-blind trial	Increase in bifidobacteria during NFSM intake.<br>Increase in bifidobacteria and lactobacilli and clostridia decreased during FSM intake.<br><br>Decrease in unclassified Clostridiaceae<br>Firmicutes decreased for both soy-milk groups (LGS and S)	Decrease in Lactobacillus<br><br>Decrease in bacterial diversity and richness (ACE and Chao1 indices) in all arms while unchanged in the M group<br><br>Bifidobacterium decreased within LGS and S and increased within the M group	No change in alpha diversity & in beta-diversity

**Specific foods- Legumes_nuts** (n=15)	**No of studies**	**Type of design**	**Beneficial effect**	**Harmful effect**	**No effect**
			Bacteroidetes increased in both soymilk groups and decreased in the M group<br>Firmicutes to Bacteroidetes ratio decreased in both the LGS and S groups and remained relatively unchanged in the M group<br>Bacteroides-Prevotella increased significantly in LGS but not S or M<br>Faecalibacterium increased in LGS group	Lactobacillus increased within M but not in LGS and S<br>Roseburia tended to increase whereas Prevotella tended to decrease in bovine milk arm and opposite for S group	
Legume consumption, including peanuts, soy foods, and other beans - total legume consumption (in grams/day) - Wang_2021	1	Population-based cohort study	Enterobacteriales positively associated with high legume intake		α-diversity or β-diversity was not associated with legume consumption

Table S1.d. Summary tables – Grains

**Specific foods - Grain** (n=20)	**No of studies**	**Type of design**	**Beneficial effect**	**Harmful effect**	**No effect**
A diet high in Whole Grains (>80 g/d) or low in WGs (<16 g/d, RG diet) - Ampatzoglou, 2015<br><br>Whole grain daily (WW - 105 g) or no whole grain (RW) both in energy-restricted diet (deficit of at least 1250 kJ/day) - Christensen_2013<br><br>Whole grain oat Granola (WGO – 45g/day) or non-whole grain (NWG) - Connolly_2016<br><br>Whole grain (WG - 48 g/d, 1442 kJ/100 g, 18 g/100 g fiber) or wheat bran (WB - 48 g/d, 1184 kJ/100 g, 27 g/100 g fiber) - Costabile_2008<br><br>Durum wheat (75%) and whole-grain barley (25%) pasta containing 3 g of barely beta-glucans - 100 g/day - DeAngelis_2015	6	Crossover study<br><br>Dietary intervention, open-label parallel, randomized<br><br>A randomized, controlled, crossover, double-blinded design<br><br>A double-blind, randomised, placebo-controlled, crossover study<br><br>Dietary intervention<br><br>A placebo-controlled,	Increase in abundance of Bifidobacterium after whole grain daily<br>Decrease in abundance of Bacteroides after no whole grain diet<br><br>Increase in bifidobacterial, lactobacilli, and total bacteria population after WGO<br><br>Increase in bifidobacteria during the ingestion of the WG<br>Increase in fecal lactobacilli/enterococci with ingestion of either WB or WG<br><br>Increase in SCFA (2-methyl-propanoic acid, acetic acid, butanoic acid, and propanoic acid) after the intervention<br>Increase in Roseburia hominis<br><br>Increase in Prevetolla after WG intake<br>Decrease in Dialister, Blautia, and	Decrease in bifidobacteria and total bacteria population after NWG<br><br>Increase in clostridia during WB intake<br><br>Fewer total anaerobes, decrease in Firmicutes after the intervention<br><br>Increase in Clostridiaceae (Clostridium orbiscindens and Clostridiumsp.), and Ruminococcus sp<br><br>Decrease in Bifidobacterium	No significant change in SCFA or bacterial groups<br><br>No significant changes in SCFA<br><br>No change in Chao1 and Shannon index<br><br>No change at phyla level<br><br>No change in α-diversity or β-diversity

**Specific foods - Grain** (n=20)	**No of studies**	**Type of design**	**Beneficial effect**	**Harmful effect**	**No effect**
Whole grain (WG) product - 70 g/d(3 biscuits/d) VS Control - 1 package (33 g) of crackers and 3 slices of toasted bread (˜27 g) Vitaglione_2015 (C)		parallel-group randomized trial	Collinsella after WG intake		
Whole grain market basket -13.7 g of fiber/day VS refined grain market basket - 4.2 g of fiber/day - Cooper_2017<br><br>Whole grain diet (≥75g/day whole grain) VS a refined grain diet (<10g/day whole grain) - Roager_2019<br><br>Whole grain wheat (WGW) (98 g/d - 17.6 g fiber/100 g) VS colored refined wheat (RW) products (98 g/d - 7.2 g fiber/100 g) - vanTrijp_2021<br><br>A whole-grain (WG) diet (16 g/1000 kcal - 35 g/d) VS refined grains (RGs) diet (8 g/1000 kcal) - Vanegas_2017	4	A six-week intervention trial<br><br>A randomized, controlled crossover<br><br>Double-blind, randomized, controlled, parallel<br><br>Randomized, controlled, parallel-design human trial	Increase in relative abundance of Akkermansia and Lactobacillus in the high fiber group<br><br>Increase in Faecalibacterium prausnitzii, one Prevotella copri after whole grain<br><br>Decrease in Bacteroides thetaiotaomicron after whole grain<br><br>Decreased in bacterial taxon within the Lachnospiraceae family after WGW diet<br><br>Increase in acetate and total SCFA in the WG<br><br>Decrease in Enterobacteriaceae after WG<br>Increase in Roseburia after WG	Increase in Clostridiales after whole grain<br><br>Increased 3 bacterial taxa within the Ruminococcaceae family) after WGW diet<br><br>Increase in Lachnospira after WG	No significant difference in the relative abundance of any taxa<br><br>No change in richness or evenness<br><br>No change in α-diversity or β-diversity<br><br>No change in α-diversity or β-diversity<br><br>No change at phyla level
Intervention arm - Rye breads with a high-fiber content (7–15%), whole-meal pasta [3.5 dL/wk and high-fiber oat biscuits VS controls - refined white wheat breads with	4	A randomized, parallel, 2-arm 12-wk intervention	Increase in Actinobacteria, Bifidobacterium, Bifidobacteriales,	Decrease in Bacteroidetes phylum and increase in the members of Clostridium	No change in microbiota composition

**Specific foods - Grain** (n=20)	**No of studies**	**Type of design**	**Beneficial effect**	**Harmful effect**	**No effect**
a low fiber content (4%) and restricted rye intake to 1–2 portions/d.- Lappi_2013<br><br>Whole-grain wheat (WGW) VS Whole-grain rye (WGR), or Refined wheat (RW) - Vuholm_2017<br><br>White bread period VS a white rice period - Mano_2018<br><br>Brown rice flakes 60g (4.4g total dietary fiber) VS Brown rice and barley (consisting of 30 g each - 11.5 g total dietary fiber) VS Barley (consisting of 60 g - 18.7 g total dietary fiber) - Martínez_2013		A randomized parallel researcher-blinded parallel intervention study<br><br>Randomized, crossover trial<br><br>Randomized cross-over trial	Bifidobacteriaceae after bread period compared to rice period<br><br>Increase in community evenness (Shannon's and Simpson's), after all interventions<br><br>Increase in Roseburia, Bifidobacterium and the species E. rectale after intake of diets containing barley	cluster IV (Firmicutes) in the control arm<br><br>Butyrate decreased in in the refined wheat arm<br><br>Firmicutes increased and bacteroidetes decreased in all three dietary treatments<br><br>Increase in Firmicutes/Bacteroidetes ratio<br>Increase in Dialister	No change in the relative abundance of any bacterial taxa after the intervention arm<br><br>No changes in alpha- and beta-diversity<br><br>No change at genera level<br><br>No change in Bacteroidetes and Firmicutes<br><br>No significant change in SCFA
Oatmeal porridge- 60 g/d oatmeal (contained per 100 g - 8.5g dietary fiber, including 4.7g β-glucans - Valeur_2016<br><br>Oatmeal - 80 g/day VS refined white rice 80 g/day - Ye_2020	2	Pilot study - dietary intervention<br><br>A secondary analysis of a randomized, controlled clinical trial		Decrease in the relative abundance of Bacteroides phylum (and Proteobacteria phylum in the oatmeal arm<br>Increase in the relative abundance of Firmicutes phylum in the oatmeal arm	No significant change in SCFA<br><br>No changes in alpha- and beta-diversity
Himalaya 292 (96g/d - a novel hull-less barley - higher total dietary fiber) VS whole-wheat (97 g/d) VS refined cereal foods (99 g/d) - Bird_2008	1	A randomized cross-over design	Higher excretion of butyrate or higher faecal total SCFA excretion during the consumption of Himalaya 292<br>Excretion of anaerobes was significantly		

**Specific foods - Grain** (n=20)	**No of studies**	**Type of design**	**Beneficial effect**	**Harmful effect**	**No effect**
			higher during the consumption of Himalaya 292		
Foods produced from unrefined flour mix composed an equal percentage of "Tim-ilia", "Margherito", and "Russello" (all ancient grains) VS refined flour from "Simeto wheat - Carroccio_2021	1	A non-randomized, nutrition intervention	Increase in abundance of culturable enterococci, lactic acid bacteria (LABs) and total anaerobes after diet containing ancient grains		No change in Shannon species diversity index<br><br>No change at the high taxonomic levels, specifically phyla, families, and genera
Simple (a higher proportion of foods containing sucrose and/or high-fructose corn syrup) VS Refined (foods made from refined grains, such as white rice, white bread, and white pasta), VS Unrefined (foods made from whole grains) carbohydrate-containing foods - Faits_2020	1	A post hoc analysis of a randomized clinical trial, crossover design	Increase in Roseburia and Anaerostipes after consumption of the unrefined carbs		No change in α-diversity or β-diversity<br>No significant change in SCFA
Whole grain (WG) diet - 40g/d dietary fiber & ≤ 30g/d red meat products VS Red meat (RM) diet -200g/d - Foerster_2014	1	Randomized Crossover design	Increase in diversity after WG diet		No significant change in SCFA<br>No change at phylum level

Table S1.e. Summary tables – Fiber

**Specific foods – Fiber** (n=3)	**No of studies**	**Type of design**	**Beneficial effect**	**Harmful effect**	**No effect**
'African style' foods increasing their average fiber intake from 14 to 55 g per day and reducing their fat from 35% to 16% of total calories VS 'Western-style' diet reducing their fiber from 66 to 12 g per day and increasing their fat from 16% to 52% - Okeefe_2015	1	food exchange intervention	Increase in butyrate in colonic evacuates 2.5 times after 'Africanization' of the diet<br>High-fibre, low-fat dietary intervention in African Americans - associated with an increase of Firmicutes, and E. rectaleet rel.	Low-fibre, high-fat intervention in Africans - associated with an increase of F. nucleatum<br>High-fibre, low-fat dietary intervention in African Americans - associated with Clostridium symbiosumet rel	
dietary fiber intake 40 g/day - 50 g/day - Oliver_2021	1	dietary fiber intervention	A trend to increase in acetate, propionate, butyrate, and valerate<br>Increase in Bifidobacterium, Lactobacillus, Coprococcussp and Anaerostipes hadrus after intervention<br>Decrease in Lachnospiraceae after intervention	Decrease in alpha diversity after intervention	
High-fiber diet (increased fiber consumption to 45.1±10.7 g /day) or High-fermented-foods diet (increased consumption to an average of 6.3±2.9 servings/day) - Wastyk_2021	1	randomized, prospective study	Increase in microbiota diversity after high-fermented-food diet	Increase in the genus Lachnospira in the high-fiber-diet arm and a decrease in the high-fermented-food arm	No change in butyrate in high-fiber diet arm

Table S1.f. Summary tables – Fruits

**Specific foods - Fruits** (n=19)	**No of studies**	**Type of design**	**Beneficial effect**	**Harmful effect**	**No effect**
Green olives - 12 table green olives/day - Accardi_2016	1	Pilot nutritional intervention	A trend towards an increase of Lactobacilli after green olive intake		
Mango 400g/day for 6 wks - Barnes_2019	1	Clinical pilot trial	A trend toward increased levels of butyric acid and valeric acid		No significant change in SCFA<br>No change in microbiota composition at the genus level for Lactobacillus or Bifidobacterium
A dried daily portion (equivalent to one apple) of the red-fleshed or placebo (white-fleshed) apple - Barnett_2021	1	A randomized, placebo-controlled, crossover	Decrease in Streptococcus, Ruminococcus, Blautia, and Roseburia<br>Increase in Butyricicoccus, and Lactobacillus after red apple intake	Increase in Sutterella, after red apple intake	No change in alpha diversity
Sweetened dried cranberries - 42 g daily - Bekiares_2018	1	A prospective clinical study	Decrease in Firmicutes, Firmicutes:Bacteroidetes ratio<br>Increase in Bacteroidetes & Akkermansiain		
Cranberry diet (basal/animal-based diet plus 30 g/day of freeze-dried whole cranberry powder) VS control diet (basal/animal-based diet plus 30 g/day of matched placebo powder) - Rodríguez-Morató_2018	1	Randomized, double-blind, cross-over, controlled design trial	A decrease in acetic and butyric acids after animal-based diet was attenuated by cranberry powder<br>Increase in Bacteroidetes and a decrease in Firmicutes after addition of cranberries	Decrease in the gram-negative Bacteroidetes and an increase in gram-positive Firmicutes after animal-based diet	No changes in alpha diversity and beta diversity

**Specific foods - Fruits** (n=19)	**No of studies**	**Type of design**	**Beneficial effect**	**Harmful effect**	**No effect**
Orange juice from Cara Cara or Bahia juices (500ml/day) or an isocaloric control drink containing water sucrose, and vitamin C - Brasili_2019<br><br>Orange juice 300 mL/day - Cesar_2020<br><br>Flavonoid-rich orange juice (190mL each 600 ±5.4 mg flavonoids 2 times/day) VS Flavonoid-low orange flavored cordial drink (190mL each 108 ±2.6 mg flavonoids 2 times/day) - Park_2020	3	A randomized, crossover and controlled trial<br><br>A controlled nonrandomized clinical study with temporal series intergroup design<br><br>A Randomized Controlled single-blind Study	Increase of Lactobacillus spp., Bifidobacterium spp and Total anaerobic bacteria	Increase in Clostridia OTUs after orange intake<br><br>Increase in Lachnospiraceae family after intake flavonoid-rich orange juice	No change in Clostridium spp
Dates (7/day - 50 g) VS a control group (maltodextrin–dextrose, 37.1 g) - Eid_2015	1	A randomized, controlled, cross-over intervention			No changes in SCFA<br>No significant alterations in faecal microbiota
Standardized freeze-dried strawberry powder (SBP) (2×13g/day) - Ezzat-Zadeh_2021	1	Intervention study			No change in α-diversity or β-diversity<br>No changes in SCFA<br>No changes on the phylum level
Avocado one/day VS no avocado - Henning_2019<br><br>Avocado 175 g (men) or 140 g (women) daily VS isocaloric control meal-Thompson_2021	2	Randomized, parallel-controlled, open-label, 2-arm intervention study<br><br>An investigator-blinded, parallel-	A trend to increase in Firmicutes after avocado intake<br><br>Increase in Veillonellaceae and Prevotellaceae after avocado intake<br><br>Increased Phylogenetic Diversity, a measure of microbiota α-diversity in the avocado group	Increases in Dialister, Sutterella, Bilophila, Holdemanella, Herbaspirillum, and Acetivibrio after avocado intake<br><br>A trend to increase for Ruminoccocaceae after avocado intake<br><br>Increase in Alistipes, Ruminococcus, and	No change in α-and β-diversity<br>No change in Bacteroidetes<br><br>No significant change in other phyla after avocado intake

**Specific foods - Fruits** (n=19)	**No of studies**	**Type of design**	**Beneficial effect**	**Harmful effect**	**No effect**
		arm, randomized controlled trial	Weighted Unifrac distances, a measure of β-diversity, tended to be greater in the avocado group<br><br>Increase in Fecal acetate in the avocado group<br><br>Increase in Faecalibacterium (P=0.07) in the avocado group<br>Decrease in Roseburia and Ruminococcus in the avocado group	Lachnospira in the avocado group	
A (poly)phenol-rich extract (116 mg, 75 g berries), a whole fruit powder (12 mg, 10 g berries), or placebo (maltodextrin) daily - Istas_2019	1	A double-blind, parallel design, randomized controlled trial	Increase in Anaerostipes after consumption of aronia extract	Increases in Bacteroides after aronia whole fruit	No changes in gut microbiota diversity
Fruits and vegetables (FV) group 3 servings/d VS whole grain (WG) servings/d VS refined grain 3 servings/d - Kopf_2018<br><br>Fruit and vegetable intake in quartiles with the lowest quartile indicating low intake - Jiang_2020	2	A randomized controlled - parallel arm feeding trial<br><br>Prospective cohort study	Increase in α-diversity in FV groups but no change in other group<br><br>Habitual fruit intake was positively associated with Observed species, Shannon index and Chao 1 index<br><br>Fruit in-take was prospectively positively associated with 31 gut microbial OTUs assigned to Faecalibacterium prausnitzii, Akkermansia	Fruit in-take was prospectively positively associated with gut microbial OTUs assigned to Ruminococcaceae, Clostridiales, Acidaminococcus, and Enterobacteriaceae.<br><br>Vegetable intake was positively associated with 1 OTU belonging to Lachnospira	No changes in SCFA<br>No significant change in bacterial genera

**Specific foods - Fruits** (n=19)	**No of studies**	**Type of design**	**Beneficial effect**	**Harmful effect**	**No effect**
			muciniphila, Prevotella stercorea, and Prevotella copri.		
One medium banana (120gm) VS one cup of banana-flavoured drink (170 ml) VS one cup of water (170 ml) - Mitsou_2011	1	A randomised, controlled trial	Increase in bifidobacterial levels in the banana group		No change SCFA
Boysenberry juice beverage (350ml/d - 750 mg polyphenols), VS apple fiber beverage (350ml/d - 7.5 g dietary fiber) VS Boysenberry juice plus apple fiber beverage (350ml/d - 750 mg polyphenols plus 7.5 g dietary fiber) VS placebo beverage - Wallace_2015	1	Placebo-controlled crossover			No change SCFA<br>No differences in fecal levels of total bacteria, Bacteroides-Prevotella-Porphyromonas group, Bifidobacterium species, Clostridiumperfringens, or Lactobacillus species
Sun dried raisins 3X28.3 g each daily - Wijayabahu_2019	1	Dietary intervention	A trend towards an increased relative abundance of Bacteroidetes and decreased relative abundance of Firmicutes<br><br>Increased in Faecalibacterium prausnitzii.<br><br>Decrease in Klebsiella spp.	Decrease in Prevotella sp. and Bifidobacterium spp<br><br>Increased in Bacteroidetes sp. and Ruminococcus sp.	No changes in alpha diversity and beta diversity
Two SUNGold Kiwifruit every day - Wilson_2018	1	Pilot intervention trial	Increase in the relative abundance of bacterial family Coriobacteriaceae		No changes in alpha diversity

Table S1.g. Summary tables – Vegetables

**Specific foods - Vegetables** (n=10)	**No of studies**	**Type of design**	**Beneficial effect**	**Harmful effect**	**No effect**
Green leafy vegetables (GLV) - 1 cup/day (including spinach, kale, collards, mustard greens, and turnip greens) VS habitual diet (high in red meat) - Frugé_2021	1	Randomized controlled crossover trial			No changes in alpha diversity<br>No significant differences within phyla b/n groups or across time
Fresh (180 g/day) vs fermented kimchi (180 g/day) group - Han_2015<br><br>Fermented kimchi intake (100g/day) - Park_2021	2	A randomized controlled clinical<br><br>Dietary intervention	Decrease in Firmicutes/Bacteroidetes ratio in response to both groups of kimchi intervention<br><br>Increase in Actinobacteria after kimchi intervention in both group<br><br>Increase in Bacteroides and Prevotella and a decrease Blautia after intake of fermented kimchi<br>Increased in observed index, Chao1 Richness index and Shanon index after fermented kimchi intake<br><br>Decrease in Enterococcua, Coryobacteriaceau in patients with advanced colon adenoma after kimchi intake	Increase in Proteobacteria after kimchi intervention in both group<br><br>Decrease in Roseburia, Bifidobacterium spp., and Akkermansia in patients with advanced colon adenoma after kimchi intake	

**Specific foods - Vegetables** (n=10)	**No of studies**	**Type of design**	**Beneficial effect**	**Harmful effect**	**No effect**
Inulin-type Fructans-rich Vegetables (ITF-rich - artichoke, artichoke bottoms, Onion, garlic, shallot, salsify, Leek, scorzonera, gratin, Celery root) - ≥9 g/day of fructans VS habitual diet - Hiel_2019	1	A single group-design intervention	Increase in Actinobacteria phylum and class, Actinobacteridae subclass, Bifidobacteriales order, Bifidobacteriaceae family, and Bifidobacterium genus in ITF-rich veggies arm<br><br>Decrease in the Alistipes genus and Oscillibacter genus, and an increase in the Prevotellaceae family in ITF-rich veggies arm	Decrease in observed species index of richness	No change in SCFA production, at phylum and family levels
Broccoli plus base diet (200 g/day (20 g daikon radish) vs base diet (control diet – American foods) - Kaczmare_2019<br><br>Low-Brassica diet (84 g frozen broccoli, 84 g frozen cauliflower, and a single portion of the participant's choice per wk) VS High-Brassica diet (6X 84 g of frozen broccoli, 6X 84 g of frozen cauliflower and 6X300 g of a broccoli and sweet potato soup containing 84 g broccoli. - Kellingray_2017<br><br>Broccoli sprouts (20 g/day - 4.4 mg/g sulforaphane glucosinolates) Vs the alfalfa sprouts (AS) group (20 g/day – no sulforaphane glucosinolates) - Yanaka_2018	3	Randomized crossover a single-blind, design<br><br>Randomized crossover study<br><br>A placebo-controlled semi-open label intervention trial	Increase in Bacteroidetes phylum & Bacteroides genus after broccoli consumption<br>Decrease in Firmicutes after broccoli consumption<br>Increase in ratio of Bacteroidetes to Firmicutes after broccoli	Decrease in beta diversity after broccoli consumption<br><br>Decrease in sulphate-reducing bacteria, Rikenellaceae, Ruminococcaceae, Mogibacteriaceae, Clostridium and unclassified Clostridiales after increased Brassica consumption.<br><br>Decrease in Bifidobacterium after broccoli intake	No change in alpha diversity<br><br>No change gut microbiota composition<br>No changes in lactobacilli
Low-phytochemical basal diet VS Single-cruciferous diet (basal diet + 7 g	1	Randomized, crossover,controlled	Eubacterium hallii, Phascolarctobacterium		

**Specific foods - Vegetables** (n=10)	**No of studies**	**Type of design**	**Beneficial effect**	**Harmful effect**	**No effect**
cruciferous vegetables/ (kg body weight a day)) VS Double-cruciferous diet (basal diet + 14 g cruciferous vegetables/(kg body weight a day)) VS Mixed diet (basal diet + 7 g cruciferous vegetables/(kg body weight a day) + 4 g apiaceous vegetables/(kg body weight a day)) - Li_2009			faecium, Alistipes putredinis, and Eggerthella spp. were associated with cruciferous vegetable intake.		
Tomato juice (330 ml/d - provided 37.0 mg lycopene and 1.6 mg β-carotene) VS Carrot juice (330 ml/d – 27.1 mg β-carotene and 13.1 mg α-carotene) - Schnäbele_2008	1	A randomized crossover trial	No change in SCFA		
Ginger juice (20 ml/d) VS sterile 0.9% sodium chloride - Wang_2020	1	Randomized controlled crossover trial	Anti-inflammatory Faecalibacterium after ginger intake<br><br>A decreased relative abundance of the Prevotella-to-Bacteroides ratio and pro-inflammatory Ruminococcus_1 and Ruminococcus_2 after ginger intake	Decrease in evenness and in richness after ginger intake<br><br>Increase in Proteobacteria, Firmicutes-to-Bacteroidetes ratio, after ginger intake	

Table S1.h. Summary tables – Macronutrients

**Specific foods** – **Macronutrients** (n=14)	**No of studies**	**Type of design**	**Beneficial effect**	**Harmful effect**	**No effect**
**Protein**<br>Very-low-calorie ketogenic diets (VLCKD-780 kcal/day) with whey protein VS vegetable protein VS animal protein - Basciani_2020<br><br>A moderately high-protein diet (MHP - 40% energy from carbohydrates, 30% from proteins, and 30% from lipids) VS A low fat diet (LF - 60% of total energy from carbohydrates, 18% from proteins, and 22% from lipids) - Cuevas-Sierra_2021<br><br>Calorie-restricted high protein diet (HPD)(30% calorie intake) VS Calorie-restricted normal protein diet (NPD) (15% calorie intake) - Dong_2020<br><br>Recommended dietary intake (RDA) of protein (0.8 g protein/kg body weight per d) VS 2RDA (1.6 g protein/kg body weight per d) - Mitchell_2020<br><br>High-protein and low-carbohydrate (HPLC; 29% protein, 5% carbohydrate, and 66% fat as calories) diet VS High-protein and moderate-carbohydrate (HPMC; 28% protein, 35% carbohydrate, and 37% fat) diet - Russell_2011	5	A prospective, open, nutritional intervention pilot study<br><br>Weight loss intervention study<br><br>A dietary intervention trial<br><br>A Parallel-Group, Randomized, Controlled Study<br><br>A randomized, crossover design, 9-wk dietary intervention study	Higher reduction in Firmicutes in whey protein and vegetable protein<br>Higher increase in Bacteroidetes after whey protein<br><br>Decrease in Negativicutes, Selenomonadales, Acidaminococcus, Bacteroides clarus, Bifidobacterium adolescentis in both diets<br>Increase in Peptococcaceae in both diets<br><br>Higher Shannon index in HPD<br><br>Decreased in Lachnospiraceae_UCG-004 in the HPD<br>Gemella is enriched in the HPD<br>Increase in Branched-chain fatty acids (BCFAs) isovalerate and isobutyrate after both diets	Decrease in the relative abundance of Firmicutes and Actinobacteria & an increase in Bacteroidetes and Proteobacteria after all VLCKD diets<br><br>Effect on diversity depend on sex<br>A minor trend of increasing Firmicutes and decreasing Bacteroides in both diets<br><br>Decreased in Prevotella_2, Faecalibaculum in the HPD<br><br>Decrease in total SCFA, butyrate after the HPLC diet<br><br>Decrease in total bacteria, Bacteroides, Roseburia/Eubacterium rectale group of butyrate producers with the HPLC diet	no change in beta-diversity<br><br>No change in α-diversity and β-diversity

**Specific foods** – **Macronutrients** (n=14)	**No of studies**	**Type of design**	**Beneficial effect**	**Harmful effect**	**No effect**
**Fat**<br>Diet with high monounsaturated fat/high glycemic index VS Diet with high monounsaturated fat/low glycemic index VS Diet with high carbohydrate/high glycemic index VS Diet with high carbohydrate/low glycemic index VS Diet with high saturated fat/high glycemic index (control diet) - Fava_2013	6	Dietary intervention study	Increase in acetate, propionate, and n-butyrate only after the high saturated fat (control diet)<br>Increase in Bifidobacterium after high carbohydrate diets<br>Increase in Bacteroides after high carbohydrate/high glycemic index diet.<br><br>Increase in Faecalibacterium prausnitzii after high carbohydrate/low glycemic index diet and high saturated fat diets	Decrease in total bacterial numbers in the high monounsaturated fat diets	
Low-calorie low-carbohydrate high-fat weight loss diet (WLD - diet plan with energy restriction of 30±10%) - Jaagura_2021		Dietary intervention study		Decrease in Bifidobacteriaceae<br>Increase in Enterobacteriaceae, Rikenellaceae,Desulfovibrionaceae<br><br>Increase in Lachnospira, Dialister and an undefined genus of Clostridiaceae, Anaerotruncus after low-fat diet	No change in alpha diversity
Low-fat diet - 50-60% of carbohydrates, 15-20% of protein per day, and maintain the intake of fat at a level of <25% - Liu_2020		long-term dietary intervention	Increase in Acinetobacter, Roseburia after low-fat diet		No change in at the phylum level
Lower-fat diet (fat 20% energy and carbohydrate 66% energy) VS Moderate-fat diet (fat 30% energy and carbohydrate		Randomized controlled-feeding trial	Increase in α-diversity after lower-fat diet		

**Specific foods** – **Macronutrients** (n=14)	**No of studies**	**Type of design**	**Beneficial effect**	**Harmful effect**	**No effect**
56% energy) VS Higher-fat diet (fat 40% energy and carbohydrate 46% energy) - Wan_2019			Decrease in Firmicutes after higher-fat diet<br>Decrease in ratio of Firmicutes to Bacteroidetes after moderate-fat and high-fat diet<br>Increase in Faecalibacterium with lower-fat diet		
Diets rich in saturated fat (SAT, 59E% fat), VS rich in unsaturated fat (UNSAT, 60E%fat), VS rich in simple sugars (CARB, 24E% fat), all diets were excess of 1000 kcal/day - Jian_2021		A randomized trial	Increase in Lactococcus and Escherichia coli after CARB<br>Increase in butyrate producers and Roseburia after UNSAT	Increase in Proteobacteria after SAT diet<br>Increase in Lachnospira and unclassified Ruminococcaceae after UNSAT	No change in alpha diversity or Firmicute to Bacteroidetes ratio
Overfeeding program - High-fat diets (HFDs) - 48 energy percent (EN%) from fat (mainly SFAs - whipping cream (341 mL/d)), 34 EN% from carbohydrates, and 18EN% from protein; a surplus of 1000 kcal/d - Ott_2018		A single-arm, prospective intervention study			No change in α-diversity & β-diversity<br>No change in fecal bacterial populations after HFD
**Carbohydrates**<br>Very low-carbohydrate, high-fat diet- (LC, 35 % of total energy as protein, 61 % as fat and 4 % as carbohydrate) VS High-carbohydrate, high-fiber, low-fat diet- (HC, 24 % as protein, 30 % as fat and 46 % as carbohydrate) - Brinkworth_2009	2	A Parallel-Group, Randomized, Controlled Study	Fecal total SCFA concentration was positively correlated with the intake of fiber and carbohydrate<br>Faecal bifidobacteria levels decreased in the LC group<br><br>Increase in proportions of branched chain	Decrease in acetate, butyrate, and total SCFA concentration in the LC group<br>Total fecal anaerobes increased in the HC group<br><br>Decrease in alpha diversity, richness, and evenness of the microbiota after the RS diet	No change in fecal propionic concentrations<br>No change in lactobacilli or anaerobe:aerobe ratio

**Specific foods** – **Macronutrients** (n=14)	**No of studies**	**Type of design**	**Beneficial effect**	**Harmful effect**	**No effect**
Diet high in type 3 resistant starch (RS) VS diet high in non-starch polysaccharides (NSPs)<br>- Salonen_2014		Randomized cross-over design	SCFA (isobutyrate and isovalerate) after RS diet	Decrease in total SCFA, acetate, propionate and butyrate in RS diet<br>Increase in Ruminococcaceae phylotypes on the RS diet<br>Increase in Lachnospiraceae phylotypes on the NSP diet	
**Multiple macronutrients and other nutrients**<br>Diet records from 24 hr dietary recall - Carrothers_2015	1	Prospective, longitudinal investigation	Higher Spirochaetes is associated with higher protein intake<br>Higher Firmicutes is associated with high n–3, n–6 FAs and higher beta-carotene<br>Higher Faecalibacterium is associated with higher insoluble fiber intake	Higher Firmicutes and lower Bacteroidetes associated with higher Carbohydrate, protein intake	

Table S1.i. Summary tables – Oils and seasonings

**Specific foods – Oils and seasonings** (n=5)	**No of studies**	**Type of design**	**Beneficial effect**	**Harmful effect**	**No effect**
14% (0.425 g) of the ADI for aspartame and 20% (0.136 g) of the ADI for sucralose - Ahmad_2020	1	Randomized, double-blind crossover and controlled clinical trial			No change in richness and evenness, SCFA, bacteria phyla and genius-level taxa
No spices VS Low spices - a mixture of 7 dried spice powders at doses 6 g VS high spices- 12g spices<br>Polyphenol-rich mixed spices - turmeric, cumin, coriander, amla (Indian gooseberry), cinnamon, clove, and cayenne pepper - KhineWWT_2021	1	Randomized, crossover, acute, food based intervention trial	Decrease in Bacteroides was with increasing spice doses<br>Increase in Bifidobacterium with increasing spice doses		No change in alpha-diversity
Daily containing 25 mL of one of the test oils: soybean oil, extra virgin olive oil or coconut oil - NettoCândido_2021	1	Randomized, parallel, double-blind clinical trial	Increase in microbial richness (Chao 1 index) after the soybean oil		No change in Shannon and Simpson indices, in beta-diversity, in SCFA<br>No change at phyla level, in Firmicutes/Bacteroidetes ratio
50 g Extra Virgin Olive Oil (EVOO) daily - Olalla_2019<br><br>Mediterranean Diet (MD) rich in High Quality-Extra Virgin Olive Oil (HQ-EVOO) - (55–60% carbohydrates, mainly complex ones, 25–30% polyunsaturated and monounsaturated fats, 15–20% proteins and received a low-calorie MD (Kcal 1,552 ± 160). Utilized 40 g/die of HQ-EVOO - Luisi_2019	2	Experimental single arm open study<br><br>Dietary intervention	Increase in Gardnerella, and a decrease of Mogibacterium, Dethiosulfovibrionaceae, and Coprococcus after intervention<br><br>Increase in Bulleidia moorei and a decrease of the Bacilli species after intervention<br><br>Increase in Lactic Acid Bacteria after the intervention		No change alpha diversity, beta diversity

Table S1.j. Summary tables – Coffee and Tea

**Specific foods - Coffee and Tea** (n=3)	**No of studies**	**Type of design**	**Beneficial effect**	**Harmful effect**	**No effect**
A single-dose serving of sugar free black coffee (30ml; ~8g Coffea arabica ground coffee powder) - Chong_2020<br><br>Coffee (3 cups/day, 3.4 g of instant coffee powder) - Jaquet_2009	2	A non-randomized, nutrition intervention<br><br>A single group-design intervention	Increase in Prevotella and reduction in Bacteroidetes after coffee intake<br><br>Increase in Bifidobacterium spp. after coffee intake		 <br>No change in diversity after coffee intake
Green tea liquid (GTL) (400 mL per day) - Yuan_2018	1	Intervention study	Increase in α-diversity & β-diversity with GTL<br>Increase in Firmicutes and Actinobacteria, and Increase in Bifidobacterium and Bifidobacterium to Enterobacteriacea ratio<br>Decrease in Bacteroidetes, Prevotellaceae, proinflammatory Fusobacterium genus, & uncultured Prevotella 9<br>Increase in SCFA-producing Lachinospiraceae, Ruminococcaceae, Erysipelotrichaceae, Bifidobacteriaceae and Coriobacteriaceae<br>Increase in SCFA-producing Roseburia, Feacalibacterium, Eubacterium, Blautia, Coprococcus, and Dorea		Increase in Firmicutes to Bacteroidetes ratio (FIR:BAC)

Table S1.k. Summary tables – Alcohol and wine

**Specific foods - Alcohol and wine** (n=2)	**No of studies**	**Type of design**	**Beneficial effect**	**Harmful effect**	**No effect**
Red wine 250 mL/day (439.5 mg of equivalents of polyphenols per day) VS no wine - Belda_2021	1	Randomized and controlled trial		Less dispersion in beta-diversity in the wine arm	No change in alpha-diversity in the wine arm<br>No significant changes in the relative abundance of Firmicutes, Bacteroidetes, Actinobacteria, Proteobacteria, Euryarchaeota, Verrucomicrobia phyla, Bacteroidetes/Firmicutes ratio
De-alcoholized red wine (272 mL/d), red wine (272 mL/d), or gin (100 mL/d) - Queipo-Ortuño_2012	1	Randomized, crossover, controlled intervention	Increase in Firmicutes and Bacteroidete after red wine intake	Increase in Proteobacteria, and Fusobacteria after red wine intake<br>Increased in Fusobacteria after de-alcoholized red wine	
